# XBP1‐elicited environment by chemotherapy potentiates repopulation of tongue cancer cells by enhancing miR‐22/lncRNA/KAT6B‐dependent NF‐κB signalling

**DOI:** 10.1002/ctm2.1166

**Published:** 2023-01-13

**Authors:** Xiaoting Jia, Ge Wang, Lihong Wu, Hao Pan, Li Ling, Jianlei Zhang, Qingquan Wen, Jie Cui, Zhimin He, Bin Qi, Shuxu Zhang, Liyun Luo, Guopei Zheng

**Affiliations:** ^1^ Affiliated Cancer Hospital & Institute of Guangzhou Medical University Guangzhou Municipal and Guangdong Provincial Key Laboratory of Protein Modification and Degradation The State Key Laboratory of Respiratory Guangzhou Guangdong China; ^2^ Affiliated Stomatology Hospital of Guangzhou Medical University Institute of Oral Disease Guangzhou Medical University Guangzhou Guangdong China; ^3^ Department of Periodontics & Oral Mucosal Section Xiangya Stomatological Hospital & Xiangya School of Stomatology & Hunan Key Laboratory of Oral Health Research Central South University Changsha China

**Keywords:** cytotoxic therapy, endoplasmic reticulum stress, NF‐κB signalling, tongue cancer, tumour repopulation

## Abstract

**Background:**

Tumour repopulation initiated by residual tumour cells in response to cytotoxic therapy has been described clinically and biologically, but the mechanisms are unclear. Here, we aimed to investigate the mechanisms for the tumour‐promoting effect in dying cells and for tumour repopulation in surviving tongue cancer cells.

**Methods:**

Tumour repopulation in vitro and in vivo was represented by luciferase activities. The differentially expressed cytokines in the conditioned medium (CM) were identified using a cytokine array. Gain or loss of function was investigated using inhibitors, neutralising antibodies, shRNAs and ectopic overexpression strategies.

**Results:**

We found that dying tumour cells undergoing cytotoxic therapy increase the growth of living tongue cancer cells in vitro and in vivo. Dying tumour cells create amphiregulin (AREG)‐ and basic fibroblast growth factor (bFGF)‐based extracellular environments via cytotoxic treatment‐induced endoplasmic reticulum stress. This environment stimulates growth by activating lysine acetyltransferase 6B (KAT6B)‐dependent nuclear factor‐kappa B (NF‐κB) signalling in living tumour cells. As direct targets of NF‐κB, miR‐22 targets KAT6B to repress its expression, but long noncoding RNAs (lncRNAs) (XLOC_003973 and XLOC_010383) counter the effect of miR‐22 to enhance KAT6B expression. Moreover, we detected increased AREG and bFGF protein levels in the blood of tongue cancer patients with X‐box binding protein‐1 (XBP1) activation in tumours under cytotoxic therapy and found that XBP1 activation is associated with poor prognosis of patients. We also detected activation of miR‐22/lncRNA/KAT6B/NF‐κB signalling in recurrent cancers compared to paired primary tongue cancers.

**Conclusions:**

We identified the molecular mechanisms of cell death‐induced tumour repopulation in tongue cancer. Such insights provide new avenues to identify predictive biomarkers and effective strategies to address cancer progression.

## 1 BACKGROUND

Cancer incidence and mortality are increasing rapidly worldwide due to complex reasons. Cancer‐related death ranks as the leading cause of death worldwide in the 21st century.^1^ Clinically and biologically, the ultimate ideal goal of cancer treatment is to remove all the cancer cells in the patient's body. Cytotoxic therapy, including chemotherapy and radiation therapy, provides clinical benefits and contributes to the long‐term survival of many cancer patients.^2^, ^3^ Generally, cytotoxic therapy induces substantial cell death, and dying or dead cells are eliminated by scavenger cells in the vicinity, leading to tumour shrinkage. However, several studies have indicated that the therapeutic benefits of cytotoxic therapy may be limited by the tumour‐promoting effects of dying or dead cells.^4^ The few surviving tumour cells escaping death during cytotoxic therapy often repopulate between treatment intervals to re‐establish tumours. The process initiated by residual tumour cells is defined as tumour repopulation, which was originally described more than 50 years ago, and finally results in cancer treatment failure.^5^, ^6^, ^7^

Recently, investigators have made many efforts to explore the mechanisms for tumour repopulation during/after chemoradiation. For example, in radiation‐induced dying tumour cells, cleaved caspase‐3 activates phospholipase A2 group VI (iPLA‐2) to enhance prostaglandin E2 (PGE2) secretion, which subsequently promotes the proliferation and growth of surviving tumour cells.^8^ In another study using a patient‐derived tumour xenograft (PDX) model, PGE2 from dying tumour cells promoted the repopulation of neighbouring cancer stem cells, conferring chemoresistance in bladder cancer. Blocking PGE2 signalling preclinically abrogated bladder cancer chemoresistance.^9^ In colon cancer, caspase‐3/7 activation induced by radiotherapy in dying tumour cells activated protein kinase C delta (PKCδ)/Akt signalling and then promoted vascular endothelial growth factor‐A (VEGF‐A) secretion to stimulate surviving tumour cell proliferation and repopulation in vitro and in vivo.^10^ Based on the literature reports, cytotoxic therapy effectively induces substantial tumour cell death and antitumour immune responses to generate clinical benefits. However, dying cells after cytotoxic treatment also establish a tumour microenvironment mainly in a secretory manner to promote immunosuppression,^11^, ^12^ tumour angiogenesis,^13^ surviving tumour cell proliferation^8^, ^14^ and metastasis,^4^ and then mediate tumour repopulation and recurrence. Owing to the efforts from many investigators, some secretory factors responsible for tumour repopulation from dying cells after cytotoxic therapy have been identified.^6^ However, the detailed mechanisms of dying cells and surviving receptor cells remain unclear.

Oral cavity cancer constitutes a broad range of tumours worldwide and was estimated to be responsible for 373 713 incident cases in 2020, accounting for approximately 2.0% of all cancer cases and is predicted to rise rapidly.^15^ Tumour repopulation and recurrence with unclear mechanisms after clinical therapy are still the major obstacles for successful treatment of oral cavity cancer. In this study, we confirmed the hypothesis that dying tumour cells after cytotoxic treatment provide initial signals in a secretory manner and clarified the detailed mechanisms for the release of tumour‐promoting factors in dying cells and for tumour repopulation in surviving tongue cancer cells.

## 2 METHODS

### 2.1 Reagents and antibodies

Cisplatin (cDDP), vitamin E (VitE) and hydralazine (Hlz) were obtained from Sigma (St. Louis, MO, USA) and 4μ8cα was purchased from Millipore (Burlington, MA, USA). MK2206, an AKT inhibitor, was purchased from Selleck (Shanghai, China). Intracellular reactive oxygen species (ROS) were detected via 2′,7′‐dichlorofluorescin diacetate (DCFDA) staining (Thermo Scientific, Waltham, MA, USA). Moreover, 4,4‐difluoro‐1,3,5,7,8‐pentamethyl‐4‐bora‐3a,4a‐diaza‐s‐indacene (BODIPY 493/503) was obtained from Thermo Scientific, and 4‐hydroxy‐trans‐2‐nonenal (4‐HNE)‐protein adducts in the indicated cells were detected through competitive enzyme‐linked immunosorbent assays (ELISAs) (Abcam, Cambridge, UK). Primary antibodies (Abs) against amphiregulin (AREG) (AB‐262‐NA) and basic fibroblast growth factor (bFGF) (AF‐233‐NA) were obtained from R&D Systems (Minneapolis, MN, USA).

### 2.2 Cells and cell culture

The human tongue cancer cell lines SCC‐25, SCC‐15, SCC‐9 and human embryonic kidney cells 293T were bought from ATCC (Manassas, VA, USA). The human head and neck cancer cell lines CNE2 and HNE1 were purchased from Cell Bank (Shanghai Institutes for Biological Sciences, China). These cells were cultured at 37°C in a humidified incubator (5% CO_2_) in Roswell Park Memorial Institute (RPMI) 1640 medium or Dulbecco's modified Eagle's medium (DMEM) (Gibco, Carlsbad, CA, USA) containing 10% foetal bovine serum (Invitrogen, Carlsbad, CA, USA). The short tandem repeat analysis was carried out to authenticate cells. All cell lines were negative for mycoplasma contamination.

### 2.3 Plasmids and lentiviral production

Lentivirus vectors expressing long noncoding RNAs (lncRNAs) XLOC_003973 and XLOC_010383 (pEZ‐Lv201), miR‐22‐3p (pEZX‐MR03), luciferase [driven by Cytomegalovirus (CMV) promoter], X‐box binding protein‐1 (XBP1) or lysine acetyltransferase 6B (KAT6B) gene‐specific shRNA (psi‐LVRU6GP) (the shRNA targeting sequences for XBP1 and KAT6B are 5′‐GACCCAGTCATGTTCTTCAAA‐3′, 5′‐GGAAGAACCTGTAGAAGATGA‐3′ and 5′‐GCACCAAAGGCAAGGATTTGG‐3′, 5′‐GCCTACCTGTGAGATTGAAGT‐3′) and the corresponding control plasmids were constructed and generated by GeneCopoeia, Inc. The full‐length XLOC_003973‐WT and XLOC_003973‐Mut and the full‐length XLOC_010383‐WT and XLOC_010383‐Mut fragments were inserted into the MS2bs vector, and the mutant was obtained by site‐directed mutagenesis of the miR‐22‐3p binding consensus sequence.

Cells were transfected with pBabe‐IκBα or control plasmid using Lipofectamine 3000 (Thermo Fisher Scientific). Each lentivirus was prepared using a Lenti‐Pac HIV Expression Packaging Kit (GeneCopoeia, China) following the manufacturer's instructions. Lentiviral particles were collected 48 h after transfection into 293T cells. Briefly, the target cells were seeded into six‐well plates overnight and then incubated with 400 µl of each lentiviral particle obtained from HEK‐293T cells for 24 h. Further, flow sorting analysis was performed to get single cell. Finally, 2 µg/ml puromycin was used to select stable cells for 2 weeks.

### 2.4 Clinical analysis

Eighty‐eight tongue cancer tissues and serum samples collected from tongue cancer patients who received cDDP‐based chemotherapy were selected from the Affiliated Cancer Hospital and Institute of Guangzhou Medical University. Fifteen cases of primary tongue cancer tissues and paired recurrent tissues were included in this cohort. All samples were collected with informed consent from the patients. Two pathologists independently identified the samples. The survival time of individuals with tongue cancer was evaluated from the diagnosis date to the date of last follow‐up or death.

### 2.5 Tumour xenograft model

Briefly, SCC‐25, SCC‐25/sh‐con, SCC‐25/sh‐XBP1 and SCC‐25/sh‐KAT6B cells were treated with 8 µg/ml cDDP for 24 h. Then, SCC‐25/Luc cells alone or mixed with cDDP‐treated SCC‐25 cells, SCC‐25/Luc cells mixed with cDDP‐treated SCC‐25/sh‐con or SCC25/sh‐XBP1 cells, SCC‐25/Luc/Con or SCC‐25/Luc/IκBα mixed with cDDP‐treated SCC‐25 cells and SCC‐25/Luc cells mixed with lethally cDDP‐treated SCC‐25/sh‐con or SCC25/sh‐KAT6B cells were subcutaneously injected into BALB/C athymic nude mice (4 weeks, Guangdong Medical Laboratory Animal Center, China) to generate xenograft tumours. The mice were pretreated with MK2206 (oral gavage, 120 mg/kg) or phosphate‐buffered saline (PBS) for 2 days, SCC‐25/Luc cells mixed with cDDP‐treated SCC‐25 cells were subcutaneously injected, and then, the mice were treated with MK2206 (oral gavage, 120 mg/kg) or PBS three times within 1 week for 4 weeks. The tumour growth was measured every 5 days. At the experimental end points, bioluminescent imaging was performed using an In‐Vivo Xtreme system (Bruker). Ten minutes before imaging, the mice were intraperitoneally injected with D‐luciferin (150 mg/kg, GoldBio, China). The animal studies were approved by the Institutional Animal Care and Use Committee (IACUC) of Guangzhou Medical University (GY2014‐057). Laboratory guidelines and animal care were in accordance with the IACUC protocol.

### 2.6 Bioluminescence imaging

For imaging luciferase, we used In‐Vivo Xtreme system. To monitor the growth of Luc‐labelled cells in vitro, we seeded 5 × 10^4^ cells per well into 24‐well plates overnight. Briefly, these cells underwent irradiation treatment or cDDP (8 µg/ml) treatment for 24 h. Immediately, these cells were mixed with 500 luciferase labelled cells. At 1, 3 and 5 days, the cells were incubated with PBS containing D‐luciferin (0.15 mg/ml) for 3 min. Finally, cells were imaged.

### 2.7 Lipid staining

Flow cytometry assays were conducted to determine the intracellular lipid content via BODIPY 493/503. Briefly, 2 × 10^5^ cells were stained with BODIPY 493/503 for 15 min at room temperature in the dark. The FITC channel was used to detect BODIPY 493/503 staining.

### 2.8 Measurement of ROS generation

Levels of intracellular ROS were estimated by CM‐H2DCFDA staining assays. Cells were incubated with 50 µM H_2_O_2_ (as positive controls) or 5 µM CM‐H2DCFDA for 30 min. The cells were washed with warmed PBS, followed by fluorescence detection using Ex/Em 495/525. The fluorescence intensity is proportional to the level of ROS produced by the cells.

### 2.9 Detection of 4‐HNE level

Whole cell lysates were obtained via treatment with radioimmunoprecipitation assay (RIPA) buffer. The lysates were centrifuged and the supernatant was obtained. Then, 4‐HNE levels were measured by ELISA depending on the instructions.

### 2.10 Western blot assays

Whole cell lysates were prepared in RIPA buffer (Thermo Scientific) with Halt Protease and Phosphatase Inhibitor Cocktail (Pierce Chemical, Dallas, TX, USA). A BCA Protein Assay Kit (Thermo Scientific) was used to measure the total protein concentration. Thirty micrograms of lysates was resolved by sodium dodecyl sulphate–polyacrylamide gel electrophoresis and then transferred to polyvinylidene difluoride membranes (Millipore, Burlington, MA, USA). After blocking for nonspecific binding, membranes were incubated with specific primary Abs overnight at 4°C. The following primary Abs were used: anti‐XBP1s antibody (Cell Signalling Technology, #27901, 1:1000 dilution), anti‐caspase‐3 antibody (Cell Signalling Technology, #9664, 1:1000 dilution), anti‐IκBα antibody (Cell Signalling Technology, #4812, 1:1000 dilution), anti‐phospho‐IκBα (Ser32) antibody (Cell Signalling Technology, #2859, 1:1000 dilution), anti‐CCND1 antibody (Cell Signalling Technology, #55506, 1:1000 dilution), anti‐H3K9Ac antibody (Cell Signalling Technology, #9649, 1:1000 dilution), anti‐H3K14Ac antibody (Cell Signalling Technology, #7627, 1:1000 dilution), anti‐Akt antibody (Cell Signalling Technology, #4685, 1:1000 dilution), anti‐p‐Akt antibody (Cell Signalling Technology, #4060, 1:1000 dilution), anti‐KAT6B antibody (Abcam, ab246879, 1:1000 dilution), anti‐Bcl2 antibody (Santa Cruz Biotechnology, sc‐7382, 1:500 dilution) and anti‐β‐actin antibody (Sigma, Cat. #A5316, 1:3000). Then, incubation with the specific horseradish peroxidase (HRP)‐conjugated antibody was performed: goat anti‐rabbit immunoglobulin G (IgG) (H + L) secondary antibody, HRP (Thermo Scientific, Cat. #31460, 1:5000) or goat anti‐mouse IgG (H + L) secondary antibody, HRP (Thermo Scientific, Cat. #31430, 1:5000). The signal was finally detected via ECL detection kit (Millipore).

### 2.11 Total RNA and miRNA isolation and quantitative real‐time polymerase chain reaction

Total RNAs and miRNAs were isolated with a MiRNA Kit (OMEGA) according to the manufacturer's instructions. cDNA was generated with a RevertAid First Strand cDNA Synthesis Kit (Thermo Scientific). Real‐time polymerase chain reaction (RT‐PCR) was performed using PowerUp SYBR Green Master Mix (Thermo Fisher). GAPDH was used as an internal control. miRNA cDNA was generated with the All‐in‐One miRNA First‐Strand cDNA Synthesis Kit (GeneCopoeia), and quantitative RT‐PCR (qRT‐PCR) was performed by using the All‐in‐One miRNA qPCR Kit (GeneCopoeia). The endogenous control RNU6 and miRNA sequence‐specific RT‐PCR primers were purchased from GeneCopoeia. The expression level of mRNA or miRNA was normalised to that of GAPDH and RNU6. Primer sequences are listed in Table S1.

### 2.12 ELISA

Conditioned medium (CM) from cultured cells and serum were analysed to assess AREG and bFGF levels via ELISA kits (R&D Systems) according to the manufacturer's instructions.

### 2.13 Soft agar colony formation assay

A total of 1.5 ml medium containing 10% FBS with 0.7% agar (Thermo Scientific) was seeded into six‐well plates per well as a base, and 1.5 ml medium containing 10% FBS, 0.35% agar and 1 × 10^3^ cells (with cDDP treatment for 24 h) per well was layered onto the base. After 2 weeks, the colonies might be well formed and should be counted. All assays were conducted in triplicate.

### 2.14 Plate colony formation assay

Target cells treated with cDDP for 24 h were seeded in triplicate in six‐well culture plates mixed with 5 × 10^4^ cDDP‐treated SCC‐25 cells/well and 500 SCC‐25/Luc cells/well. Cells were cultured to form colonies for almost 2 weeks, followed by fixing with methanol and then staining with 0.5% crystal violet. Finally, we photographed the plate and calculated the number of clones. All assays were conducted for at least three times.

### 2.15 Luciferase activity assays

Briefly, cells were seeded in 96‐well plates at a density of 5 × 10^3^ cells/well overnight. Five nanograms of Renilla luciferase reporter vectors (pRL‐TK) and 50 ng luciferase reporter vectors were co‐transfected into the cells via Lipofectamine 3000 (Invitrogen). After 48 h, a dual luciferase reporter assay system (Promega) was used to evaluate the luciferase activity. The luciferase values were normalised to the Renilla luciferase values.

### 2.16 Chromatin immunoprecipitation‐qPCR analysis

An EZ‐CHIP chromatin immunoprecipitation kit (Millipore) was utilised for chromatin immunoprecipitation (ChIP) assays. At the beginning, cells were cross‐linked with 1% formaldehyde. After quenching, the paraformaldehyde reaction with glycine, cells were lysed with lysis buffer containing Protease Inhibitor Cocktail II and sonicated on ice for chromatin immunoprecipitation assays. Ten percent of the supernatant was saved as the input fraction. The rest was used for immunoprecipitation with 5 µg of anti‐p65 antibody (Cell Signalling Technology, #8242), anti‐H3K9Ac antibody (Cell Signalling Technology, #9649) or anti‐rabbit IgG. DNA was purified and quantified by RT‐qPCR. IgG was included as nonspecific control. The results were calculated using % input = 2^(‐ΔCt [Ct [p65] – Ct [input]]) method. The primers for ChIP‐qPCR are shown in Table S2.

### 2.17 RNA immunoprecipitation

According to the manufacturer's instructions, RNA immunoprecipitation (RIP) experiments were accomplished with a Magna RIP RNA‐Binding Protein Immunoprecipitation Kit (Millipore, Billerica, MA). GFP antibody was used for RIP (Cell Signalling Technology, #55494). Coprecipitated RNA was estimated by qRT‐PCR assay.

### 2.18 Coimmunoprecipitation

Cell lysates were incubated with 3 µg anti‐p65 (Cell Signalling Technology, #3033) or anti‐IgG (Cell Signalling Technology, #2729) overnight at 4°C, and then these complexes were incubated with precleared protein A/G‐agarose beads (Millipore) for 2 h. The reaction products were washed with lysis buffer and subjected to western blot analysis for the potential proteins.

### 2.19 Immunohistochemistry and in situ hybridisation analysis

After formalin fixation and paraffin embedding, the tissues were deparaffinised in xylene and rehydrated in graded ethanol. For immunohistochemistry, the slides were immersed in 3% hydrogen peroxide and incubated with primary Abs overnight. The Abs included XBP1s (Abcam, ab37152, 1:20 dilution), AREG (Abcam, ab234750, 1:50 dilution), bFGF (Abcam, ab92337, 1:200 dilution), IκBα (Cell Signalling Technology, #4814, 1:100 dilution) and KAT6B (Abcam, ab246879, 1:200 dilution). Subsequently, the slides were incubated with HRP‐conjugated secondary Abs (Santa Cruz Biotechnology, sc‐2357, sc‐516102). Finally, after the application of DAB chromogen, tissue sections were stained with haematoxylin. For in situ hybridisation (ISH) analysis, fluorescence ISH (FISH) was performed in tissue sections using an ISH Detection kit (Boster). lncRNAs XLOC_003973 and XLOC_010383 and the miR‐22‐3p detection probe (Boster) were used, and the total staining processes were performed by following the manufacturer's protocol. Images were captured with Panoramic 250 FLASH (3DHistech) and assessed with Case Viewer software.

### 2.20 Cytokine assay

The culture supernatants of SCC‐15 with or without cDDP treatment were collected, and these cytokines were identified via a Human Cytokine Array (RayBiotech, Guangzhou, China) in accordance with the instructions. The cytokines with distinct expression between two groups were screened out.

### 2.21 Statistical analysis

Statistical analysis was conducted by SPSS version 16.0 and GraphPad Prism 7. The results are presented as the mean ± SEM of three or more experimental repeats. A chi‐square test was carried out to analyse the relationship between gene expression levels. Survival analysis were implemented by log‐rank tests and Kaplan–Meier analysis. Comparison of the mean between two groups was conducted by Student's *t*‐test. *p* < .05 was considered statistically significant.

## 3 RESULTS

### 3.1 Dying cells induced by cytotoxic treatment stimulate living tongue cancer cell repopulation

**FIGURE 1:**
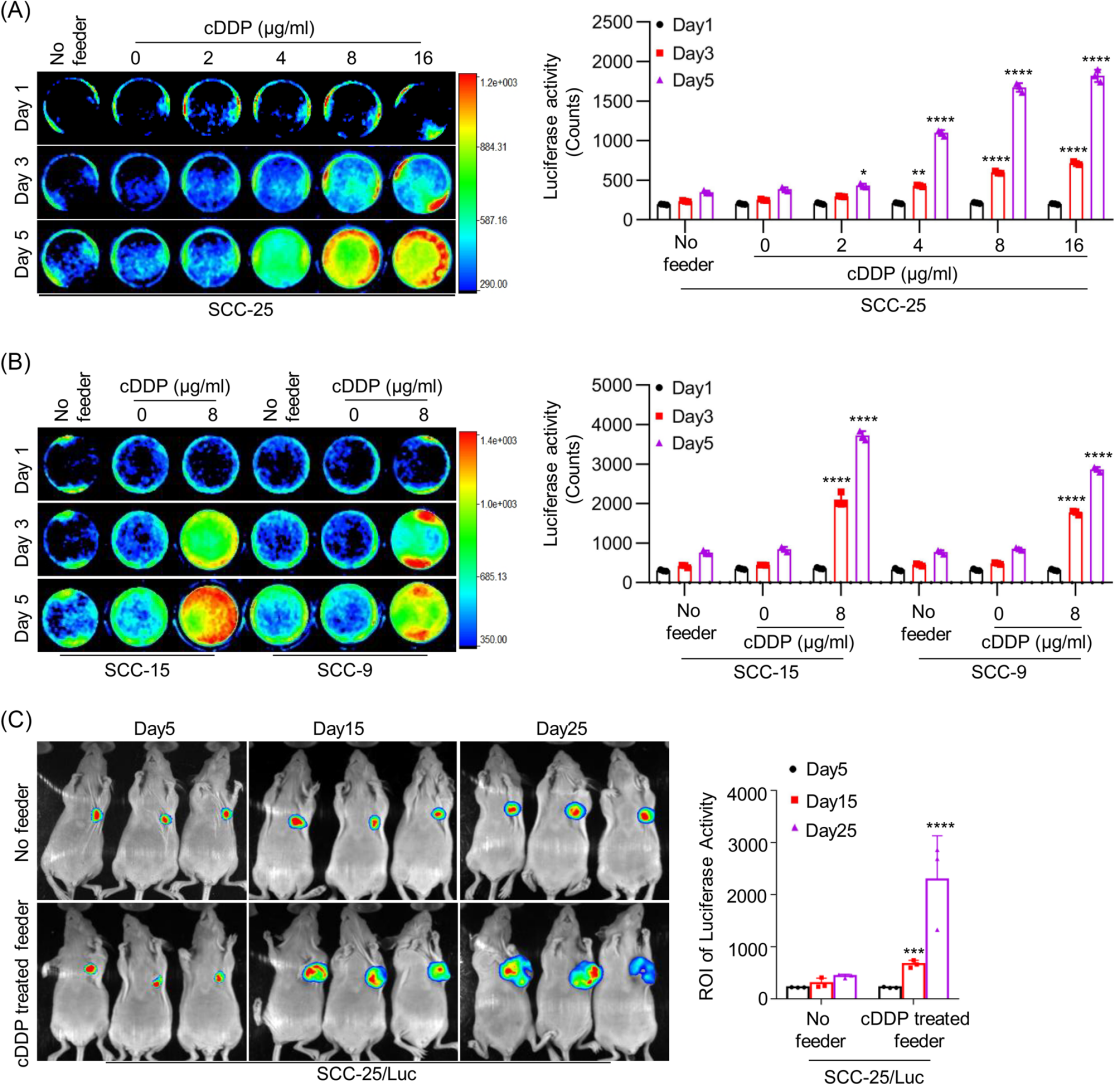
Tongue cancer cell repopulation stimulated by cytotoxic treatment‐induced dying cells. (A) SCC‐25 cells were treated with different concentrations of cisplatin (cDDP) (0, 2, 4, 8, 16 µg/ml) for 24 h in 24‐well plates, and then SCC‐25/Luc cells (reporter cells) were seeded among the SCC‐25 cells (feeder cells) with cDDP treatment or alone. The cell repopulation in vitro was observed by luciferase activities, ^*^ *p* < .05, ^**^ *p* < .01, ^****^ *p* < .0001. (B) SCC‐15 and SCC‐9 cells were treated with 8 µg/ml cDDP for 24 h in 24‐well plates, and then SCC‐15/Luc cells and SCC‐9/Luc cells (reporter cells) were seeded among each feeder cells with cDDP treatment or alone. The cell repopulation in vitro was observed by luciferase activities, ^****^ *p* < .0001. (C) SCC‐25 cells were treated with 8 µg/ml cDDP for 24 h, and then SCC‐25/Luc cells (reporter cells) were subcutaneously injected alone or together with cDDP‐treated SCC‐25 cells (feeder cells) in nude mice. The growth of SCC‐25/Luc cells is represented by luciferase levels, and the measured values (region of interest) are exhibited, ^***^ *p* < .001, ^****^ *p* < .0001. Data in (A)–(C) are represented as the mean ± SEM. Statistical significance was determined by a two‐tailed Student's *t*‐test

To explore the molecular mechanisms underlying for tumour repopulation in tongue cancer, we first investigated whether cytotoxic treatment‐induced dying tumour cells would stimulate living tumour cell repopulation in vitro and in vivo. We implemented soft‐agar colony formation and plate colony formation assays to demonstrate that cDDP dose dependently inhibited the proliferation of tongue cancer cells (Figure S1A). Consistently, cDDP dose dependently induced the activation of the apoptotic executer caspase‐3 (Figure S1B). Then, SCC‐25 cells were treated with cDDP treatment, and we called them as feeder cells. SCC‐25 cells labelled with firefly luciferase (SCC‐25/Luc) were named as reporter cells. In vitro, the SCC‐25/Luc reporter cells seeded on cDDP‐treated SCC‐25 feeder cells showed a significant increase in proliferation compared with the SCC‐25/Luc reporter cells alone or the SCC‐25/Luc reporter cells seeded on non‐cDDP‐treated SCC‐25 feeder cells (Figure 1A). Proliferation‐stimulating effects were evidently observed in the cDDP‐induced feeder cells from SCC‐15 and SCC‐9 tongue cancer cell lines (Figure 1B), as well as from two other head and neck cancer cell lines, CNE2 and HNE1 (Figure S1C). Additionally, we confirmed the effect of radiation‐induced feeder cells on tumour cell repopulation as reported previously.^8^ Radiation dose dependently inhibited the proliferation of SCC‐25 cells, as shown by colony formation and soft‐agar colony formation assays (Figure S1D), accompanied by caspase‐3 activation (Figure S1E). When compared with the SCC‐25/Luc reporter cells alone or the SCC‐25/Luc reporter cells seeded on non‐irradiated SCC‐25 feeder cells, the SCC‐25/Luc reporter cells seeded on irradiated SCC‐25 feeder cells showed a significant increase in proliferation (Figure S1F). Proliferation‐stimulating effects were also similarly observed in irradiated feeder cells from the SCC‐15, SCC‐9, CNE2 and HNE1 cell lines (Figure S1F). Moreover, death‐stimulated tumour cell repopulation was evaluated in an in vivo model. SCC‐25/Luc reporter cells alone or mixed with lethally cDDP‐treated SCC‐25 cells were subcutaneously injected into nude mice. The presence of the cDDP‐treated SCC‐25 feeder cells remarkably promoted the growth of the SCC‐25/Luc reporter cells in vivo when compared with the SCC‐25/Luc reporter cells alone (Figure 1C). These data implied that cytotoxic treatment‐induced dying cells could effectively stimulate live tumour cell repopulation in tongue cancer.

### 3.2 Cytotoxic treatment‐induced endoplasmic reticulum stress mediates tumour cell repopulation

**FIGURE 2:**
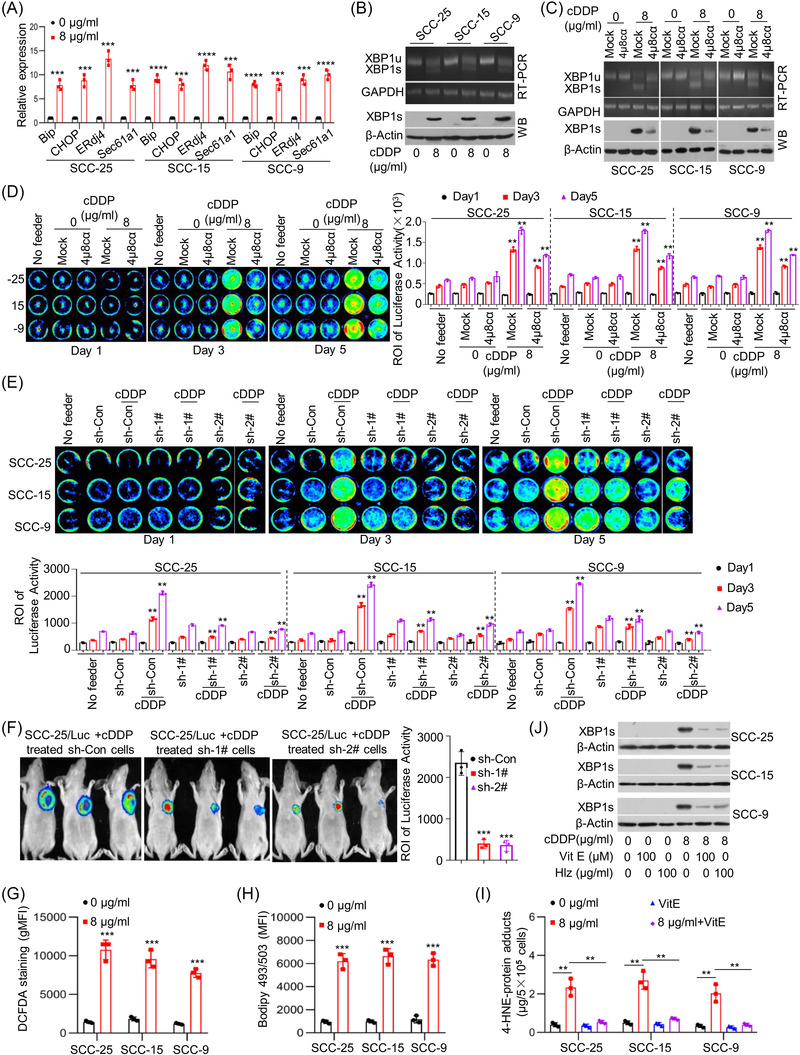
Cytotoxic treatment‐induced endoplasmic reticulum (ER) stress, mediating tumour cell repopulation. (A) SCC‐25, SCC‐15 and SCC‐9 cells were treated with cisplatin (cDDP) at the indicated concentrations for 24 h. The expression levels of BiP, CHOP, ERdj4 and Sec61a1 were measured by quantitative real‐time polymerase chain reaction (qRT‐PCR) (*n* = 3). ^***^ *p* < .001, ^****^ *p* < .0001. (B) X‐box binding protein‐1 (XBP1) splicing was evaluated using RT‐PCR (upper) and western blots (bottom). (C) SCC‐25, SCC‐15 and SCC‐9 cells were treated with vehicle (mock) or the IRE1α inhibitor 4µ8cα (10 µM) in combination with cDDP for 24 h. XBP1 splicing was evaluated using RT‐PCR (upper) and western blots (bottom). (D) SCC‐25/Luc, SCC‐15/Luc and SCC‐9/Luc reporter cells were seeded among the respective feeder cells with or without treatment with the IRE1α inhibitor 4µ8cα in combination with cDDP or alone in 24‐well plates. Cancer cell repopulation in vitro was observed by luciferase activities. ^**^ *p* < .01. (E) SCC‐25, SCC‐15 and SCC‐9 cells with or without XBP1 knockdown were treated via cDDP for 24 h in 24‐well plates. Then, SCC‐25/Luc, SCC‐15/Luc and SCC‐9/Luc reporter cells were seeded among the respective feeder cells. Cancer cell repopulation in vitro was measured by luciferase activities. ^**^ *p* < .01. (F) SCC‐25 cells with XBP1 knockdown or control vector were treated via cDDP for 24 h. Then, SCC‐25/Luc cells were injected subcutaneously together with above cells into nude mice, and the repopulation of SCC‐25/Luc cells was represented by luciferase levels, ^***^ *p* < .001. (G) The intracellular reactive oxygen species (ROS) levels in SCC‐25, SCC‐15 and SCC‐9 cells treated with cDDP were evaluated using 2′,7′‐dichlorofluorescin diacetate (DCFDA) staining (*n* = 3), ^***^ *p* < .001. (H) The intracellular lipid content in SCC‐25, SCC‐15 and SCC‐9 cells treated with cDDP was quantified using 4,4‐difluoro‐1,3,5,7,8‐pentamethyl‐4‐bora‐3a,4a‐diaza‐s‐indacene (BODIPY 493/503) staining (*n* = 3), ^***^ *p* < .001. (I) The intracellular levels of 4‐hydroxy‐trans‐2‐nonenal (4‐HNE)‐protein adducts in SCC‐25, SCC‐15 and SCC‐9 cells treated with cDDP in combination with ROS‐scavenging agent vitamin E (VitE) using enzyme‐linked immunosorbent assays (ELISAs) (*n* = 3), ^**^ *p* < .01. (J) SCC‐25, SCC‐15 and SCC‐9 cells were treated with cDDP in combination with VitE (100 µM) or 100 µg/ml hydralazine (Hlz). XBP1 splicing was detected by western blotting. Data in (A) and (D)–(I) are represented as the mean ± SEM. Statistical significance was determined by a two‐tailed Student's *t*‐test

The endoplasmic reticulum (ER) is an important subcellular organelle for the biosynthesis of proteins, lipids and so on. ER stress resulting from extracellular or intracellular challenges regulates cellular homeostasis and determines cell fate, whereas persistent ER stress triggers the apoptotic pathway.^16^ Chemotherapy, as a stress imposed on cancer cells, usually induces ER stress, resulting in cancer cell death.^17^ Adaptation to ER stress leading to resistance to ER stress‐induced cell death is positively correlated with chemoresistance. Thus, enhancing ER stress has become an attractive strategy to sensitise cancer cells to chemotherapy.^18^ Consistent with previous observations, we found that cDDP treatment induced the ER stress response in SCC‐25 cells, as indicated by dose‐dependent increases in the expression of the ER stress response markers lumenal binding protein (BiP), C/EBP homologous protein (CHOP) and spliced XBP1s and its target genes ER DnaJ family member 4 (ERdj4) and Sec61 translocon alpha 1 subunit (Sec61a1) (Figure S2A,B). The effect of cDDP treatment on inducing ER stress was further confirmed in selected cell lines (Figures 2A,B and S2C). In addition, induction of ER stress by irradiation was similarly observed in the selected cell lines (Figure S2D,E). To study the influence of ER stress response on tumour cell repopulation mediated by cytotoxic treatment, we inhibited ER stress by drug or shRNA approaches. As shown, pharmacological inhibition of inositol‐requiring enzyme 1 alpha (IRE1α)/XBP1 activation using the IRE1α inhibitor 4µ8cα effectively disrupted XBP1 activation induced by cDDP treatment (Figure 2C) and abolished the proliferation‐stimulating effect of cDDP‐treated feeder cells (Figure 2D). Moreover, to further validate the role of ER stress, we knocked down XBP1 using shRNAs (Figure S2F). XBP1 knockdown effectively repressed tumour cell repopulation mediated by cytotoxic treatment in vitro (Figures 2E and S2G) and in vivo (Figure 2F). Then, we sought to investigate the molecular event by which ER stress is triggered by cytotoxic treatment. Previous studies showed that chemotherapy‐induced elevation of ROS levels, and ROS‐induced ER stress by oxidising lipids to generate reactive byproducts such as the unsaturated aldehyde 4‐HNE.^17^, ^19^ Similarly, we found that cDDP treatment and irradiation significantly increased ROS level (Figures 2G and S3A–C) and lipid level (Figures 2H and S3D) in selected cancer cell lines. Consistent with active lipid peroxidation by ROS, cDDP treatment and irradiation sharply increased 4‐HNE‐protein adducts (Figures 2I and S3E). Then, we demonstrated that the ROS‐scavenging agent VitE disrupted the induction of 4‐HNE protein adducts by cDDP treatment and irradiation (Figures 2I and S3E). To discover the role of ROS‐lipid peroxidation in cytotoxic treatment‐induced ER stress, we applied VitE or Hlz (a hydrazine derivative sequestering reactive lipid peroxidation byproducts). Treatment of cells with either VitE or Hlz inhibited XBP1 activation and suppressed ER stress response markers by cDDP (Figures 2J and S3F). These results indicate that dying cell‐induced tumour cell repopulation is mediated via ROS‐lipid peroxidation–ER stress triggered by cytotoxic treatment.

### 3.3 KAT6B‐dependent NF‐κB signalling is responsible for living tumour cell repopulation stimulated by dying cells

**FIGURE 3:**
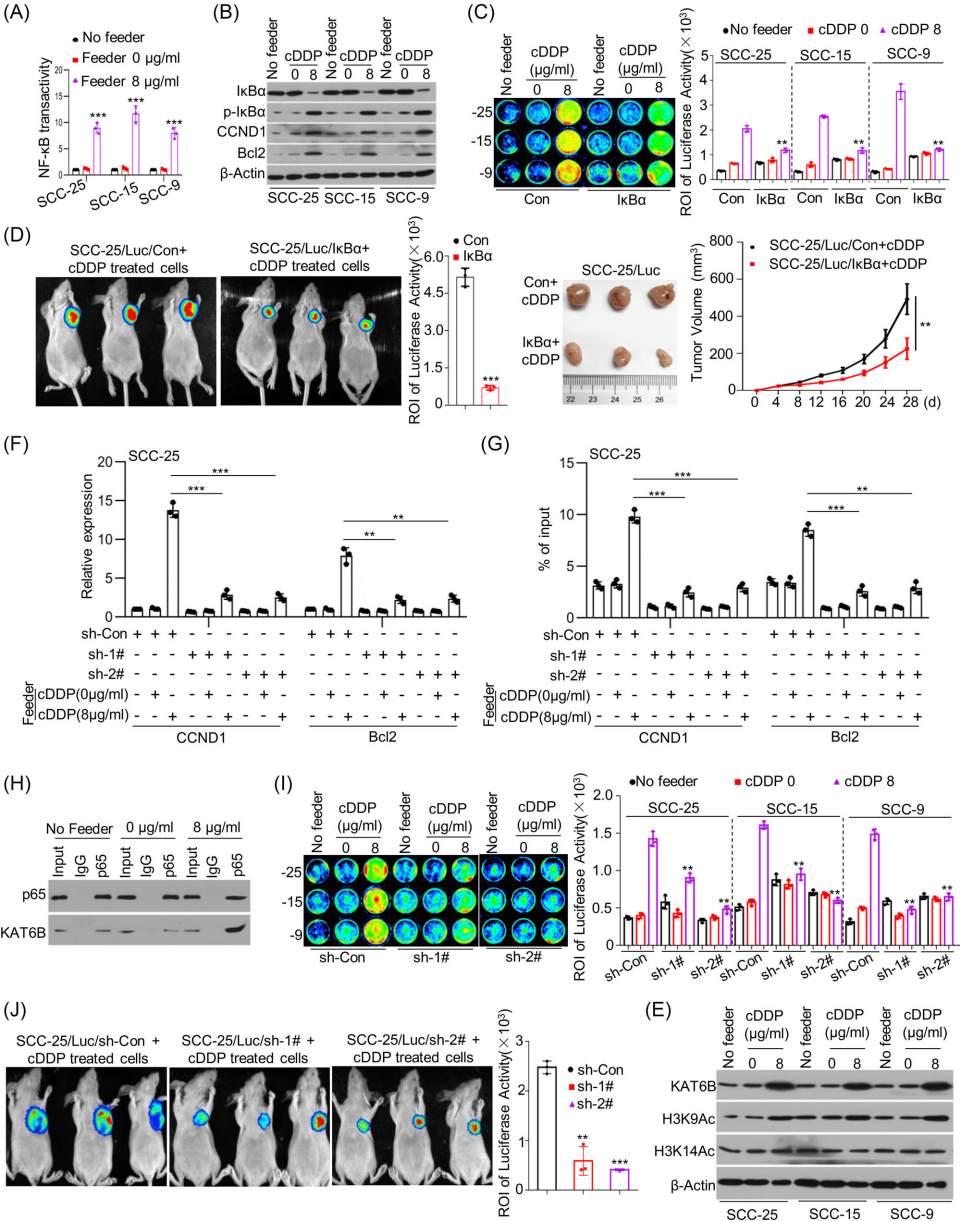
Lysine acetyltransferase 6B (KAT6B)‐dependent nuclear factor‐kappa B (NF‐κB) signalling is responsible for living tumour cell repopulation stimulated by dying cells. (A) Briefly, SCC‐25, SCC‐15 and SCC‐9 cells were treated with cisplatin (cDDP) for 24 h. Then, another SCC‐25, SCC‐15 and SCC‐9 cells were seeded among each cDDP‐treated cells. The transactivation of NF‐κB signalling in cells was measured by Cignal Reporter Assays (*n* = 3), ^***^ *p* < .001. (B) SCC‐25, SCC‐15 and SCC‐9 cells were treated with cDDP for 24 h. Then, new SCC‐25, SCC‐15 and SCC‐9 cells were seeded among each cDDP‐treated cells. The protein levels of IκBα, p‐IκBα, CCND1, Bcl2 and β‐Actin in these cells were detected by western blots. (C) SCC‐25, SCC‐15 and SCC‐9 feeder cells were treated with cDDP for 24 h. SCC‐25/Luc, SCC‐15/Luc and SCC‐9/Luc reporter cells were transfected with pBabe‐Con or pBabe‐IκBα, and then seeded among the respective feeder cells with cDDP treatment or alone in 24‐well plates. Cancer cell repopulation in vitro was evaluated by luciferase activities, ^**^ *p* < .01. (D) SCC‐25 cells were treated with cDDP for 24 h. SCC‐25/Luc reporter cells were transfected with pBabe‐Con or pBabe‐IκBα and injected subcutaneously together with cDDP‐treated SCC‐25 cells into nude mice. The growth of SCC‐25/Luc cells transfected with pBabe‐Con or pBabe‐IκBα was represented by luciferase levels and tumor size, ^**^ *p* < .01, ^***^ *p* < .001. (E) SCC‐25, SCC‐15 and SCC‐9 cells were cocultured with cDDP‐treated each feeder cells. The protein levels of KAT6B, H3K9Ac, H3K14Ac and β‐Actin were detected by western blots. (F and G) SCC‐25 cells were treated with cDDP for 24 h, another SCC‐25 cells were transfected with shRNAs specifically targeting KAT6B and then cocultured with cDDP‐treated SCC‐25 cells. The expression levels of CCND1 and Bcl2 were detected by quantitative real‐time polymerase chain reaction (qRT‐PCR) (*n* = 3) (F). The H3K9Ac levels at the promoter regions of CCND1 and Bcl2 were detected by chromatin immunoprecipitation (ChIP)‐qPCR (*n* = 3), ^**^ *p* < .01, ^***^ *p* < .001 (G). (H) SCC‐25 cells were treated with cDDP for 24 h, another SCC‐25 cells were cocultured with cDDP‐treated SCC‐25 cells. Total proteins were subjected to immunoprecipitation (IP) using an anti‐p65 antibody or control immunoglobulin G (IgG), followed by western blot analysis with a specific antibody against KAT6B. (I) SCC‐25 cells were treated with cDDP for 24 h. SCC‐25/Luc, SCC‐15/Luc and SCC‐9/Luc reporter cells were transfected with shRNAs specifically targeting KAT6B and then seeded among the respective feeder cells with cDDP treatment or alone in 24‐well plates. Cancer cell repopulation in vitro was observed by luciferase activities, ^**^ *p* < .01. (J) SCC‐25 cells were treated with cDDP for 24 h. SCC‐25/Luc cells with or without KAT6B knockdown were injected subcutaneously together with cDDP‐treated SCC‐25 cells in nude mice, and tumour growth is represented by luciferase levels, ^**^ *p* < .01, ^***^ *p* < .001. Data in (A), (C), (D), (F), (G), (I) and (J) are represented as the mean ±SEM. Statistical significance was determined by a two‐tailed Student's *t*‐test

Given that living tumour cell repopulation stimulated by dying tumour cells via cytotoxic treatment‐induced ER stress, to identify the molecular events in living tumour cells, we estimated the transactivation of several transcription factors in SCC‐25 cells after coculture with cDDP‐treated feeder cells and demonstrated that NF‐κB signalling showed the greatest activation (Figure S4A). The activity of NF‐κB signalling in living cells induced by dying cells was further validated in selected cell lines (Figures 3A and S4B). Transactivation of NF‐κB signalling was also reflected by reduced total IκBα protein levels and increased p‐IκBα protein levels and expression of NF‐κB target genes such as CCND1 and Bcl2 (Figures 3B and S4C). To investigate whether activation of NF‐κB signalling is responsible for tumour cell repopulation stimulated by feeder cells, we applied a dominant‐negative model with the pBabe plasmid and nonphosphorylatable IκBα (pBabe‐IκBα), leading to constitutive suppression of NF‐κB signalling. As shown in Figure S4D, compared with the control group, pBabe‐IκBα obviously suppressed the CCND1 and Bcl2 expression enhanced by cDDP treatment. As expected, pBabe‐IκBα transfection disrupted the promotive effect on tumour cell repopulation of living cells stimulated by dying feeder cells in vitro (Figure 3C) and in vivo (Figure 3D).

Given that NF‐κB signalling was regulated by KAT6B in tongue cancer cells, we wondered whether KAT6B was also involved in the activation of NF‐κB signalling stimulated by dying cells. We found that KAT6B expression in selected cell lines was upregulated at both the mRNA and protein levels accompanied by an increase in KAT6B target global H3K9 acetylation after culture with feeder cells receiving cytotoxic therapy (Figures 3E and S5A). To further investigate the function of KAT6B, we knocked down KAT6B expression in reporter cells (Figure S5B). KAT6B knockdown abolished the induction of NF‐κB target gene expression in reporter cells by feeder cells (Figures 3F and S5C). There was an increase in H3K9Ac levels at the p65 binding sites at the promoters of the NF‐κB target genes after coculture with the cDDP‐treated feeder cells, whereas KAT6B knockdown caused a reduction in H3K9Ac levels (Figures 3G and S5D). As expected, the coimmunoprecipitation (CoIP) assay indicated that p65 could physically interact with KAT6B (Figure 3H), suggesting that the p65 and KAT6B complex regulated histone modification at the promoter of NF‐κB target genes and promoted target gene transcription. Then, we examined the biological effect of KAT6B and found that KAT6B knockdown disrupted the promotive effect on tumour cell repopulation of living cells stimulated by dying feeder cells in vitro (Figure 3I) and in vivo (Figure 3J). These experimental results indicate that KAT6B‐dependent NF‐κB signalling activation is responsible for tumour cell repopulation by dying cells.

### 3.4 Mutual interaction between both NF‐κB‐targeted lncRNAs and miR‐22 maintains KAT6B expression

**FIGURE 4:**
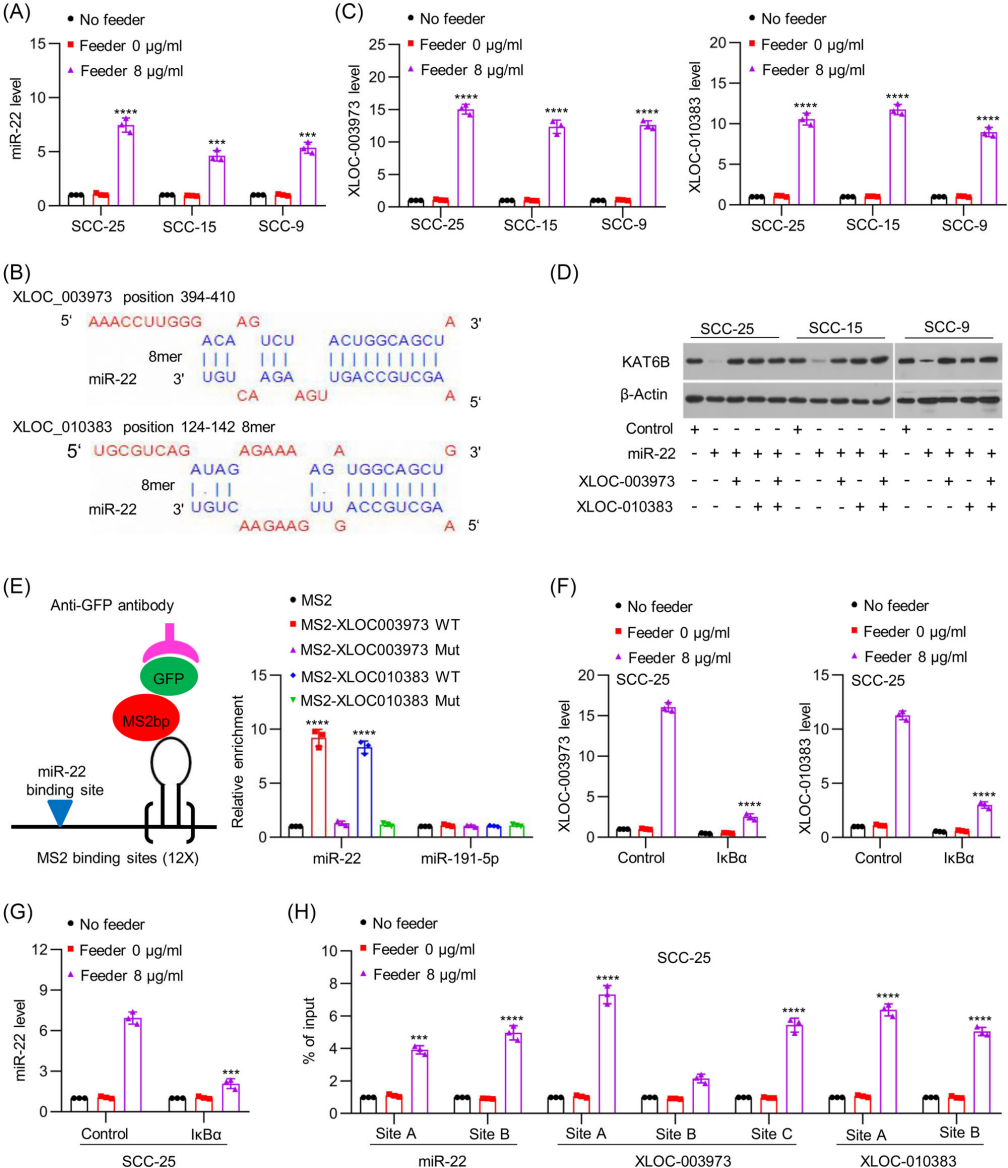
Mutual confrontation between both nuclear factor‐kappa B (NF‐κB)‐targeted long noncoding RNAs (lncRNAs) and miR‐22 maintains lysine acetyltransferase 6B (KAT6B) expression. (A) SCC‐25, SCC‐15 and SCC‐9 cells were treated with cisplatin (cDDP) for 24 h. Another SCC‐25, SCC‐15 and SCC‐9 cells were cocultured with cDDP‐treated feeder cells. The expression of miR‐22 was measured by quantitative real‐time polymerase chain reaction (qRT‐PCR) (*n* = 3), ^***^ *p* < .001, ^****^ *p* < .0001. (B) A schematic representation of the seed sequences of miR‐22 in the mRNAs of lncRNAs XLOC_003973 and XLOC_010383 predicted with DIANA tools (http://carolina.imis.athena‐innovation.gr/diana_tools/web/index.php). (C) SCC‐25, SCC‐15 and SCC‐9 cells were treated with cDDP for 24 h. Another SCC‐25, SCC‐15 and SCC‐9 cells were cocultured with cDDP‐treated feeder cells. The expression of XLOC_003973 and XLOC_010383 was measured by qRT‐PCR (*n* = 3), ^****^ *p* < .0001. (D) miR‐22 in combination with XLOC_003973 or XLOC_010383 was overexpressed in SCC‐25, SCC‐15 and SCC‐9 cells. The protein levels of KAT6B and β‐Actin were detected by western blots. (E) SCC‐25 cells were transfected with MS2bp‐GFP overexpression vector or MS2bs vectors cloned with related DNA sequences (MS2bs, MS2bs‐XLOC_003973‐WT, MS2bs‐ XLOC_003973‐Mut, MS2bs‐ XLOC_010383‐WT and MS2bs‐ XLOC_010383‐Mut). At 48 h, related cells were used to perform RIP assays using anti‐GFP antibody. After extraction of RNAs, miR‐22 and miR‐191‐5p were examined by qRT‐PCR (*n* = 3), ^****^ *p* < .0001. (F and G) SCC‐25 cells were treated with cDDP for 24 h. Another SCC‐25 cells were transfected with pBabe‐Con or pBabe‐IκBα and then cocultured with cDDP‐treated feeder cells. The expression levels of XLOC_003973 and XLOC_010383 (F) and miR‐22 (G) were measured by qRT‐PCR (*n* = 3), ^***^ *p* < .001, ^****^ *p* < .0001. (H) SCC‐25 cells were treated with cDDP for 24 h. Another SCC‐25 cells were cocultured with cDDP‐treated feeder cells. The enrichment of p65 at the promoters of miR‐22, XLOC_003973 and XLOC_010383 was evaluated by chromatin immunoprecipitation (ChIP)‐qPCR (*n* = 3). Chromatin was precipitated with an anti‐p65 antibody. The precipitated chromatin was then analysed by qRT‐PCR with primers specific for the putative p65 binding sites, ^***^ *p* < .001, ^****^ *p* < .0001. Data in (A), (C) and (E)–(H) are represented as the mean ± SEM. Statistical significance was determined by a two‐tailed Student's *t*‐test

Previously, we demonstrated that miR‐22 (miR‐22‐3p) post‐transcriptionally regulated KAT6B expression in tongue cancer cells.^20^ Here, we sought to explore whether the upregulation of KAT6B expression was due to downregulation of miR‐22 expression. Unexpectedly, we found that miR‐22 expression in living cells after coculture with dying cells was upregulated but not downregulated (Figures 4A and S6A). Therefore, we hypothesised that lncRNAs might act as competing endogenous RNAs to address the effect of miR‐22, leading to upregulating KAT6B expression. To test this possibility, we carried out bioinformatics analysis via the online tool DIANA.^21^ Two potential lncRNAs, XLOC_003973 and XLOC_010383, were predicted to interact with miR‐22 by an 8‐mer site (Figure 4B). The expression of XLOC_003973 and XLOC_010383 was significantly elevated in living cells after coculture with dying cells (Figures 4C and S6B). Then, we investigated the biological effects of miR‐22 and lncRNAs (XLOC_003973 and XLOC_010383) on the expression of KAT6B. We found that ectopic overexpression of miR‐22 effectively reduced endogenous KAT6B expression, whereas ectopic overexpression of XLOC_003973 and XLOC_010383 synergistically reversed the reduction in KAT6B expression induced by miR‐22 (Figures 4D and S6C). To validate the direct interaction between lncRNAs (XLOC_003973 and XLOC_010383) and miR‐22 via the predicted binding motif, we performed a RIP assay, and the association between lncRNAs (XLOC_003973 and XLOC_010383) and miR‐22 was confirmed (Figure 4E). Then, we wondered whether lncRNAs (XLOC_003973 and XLOC_010383) and miR‐22 were simultaneously regulated by NF‐κB signalling. Ectopic IκBα overexpression disrupted the induced expression of lncRNAs XLOC_003973 and XLOC_010383 in living cells by dying cells (Figures 4F and S6D), as well as miR‐22 (Figures 4G and S6E). To validate whether XLOC_003973, XLOC_010383 and miR‐22 were direct targets of NF‐κB, we performed bioinformatics analysis (the JASPAR database) and identified two, three and two NF‐κB binding sites at the potential promoters, respectively (Figure S6F). ChIP assays indicated the occupancy of p65 on the promoters of XLOC_003973, XLOC_010383 and miR‐22, and the occupancy was enhanced by coculture with dying cells (Figure 4H). These results indicate that NF‐κB transcriptionally promoted miR‐22 expression to suppress KAT6B expression and promoted XLOC_003973 and XLOC_010383 expression to counter the effect of miR‐22, finally leading to maintenance and enhancement of KAT6B‐dependent NF‐κB signalling.

### 3.5 Cytotoxic treatment‐induced dying cells promote tumour cell repopulation via secretion of amphiregulin and basic fibroblast growth factor

**FIGURE 5:**
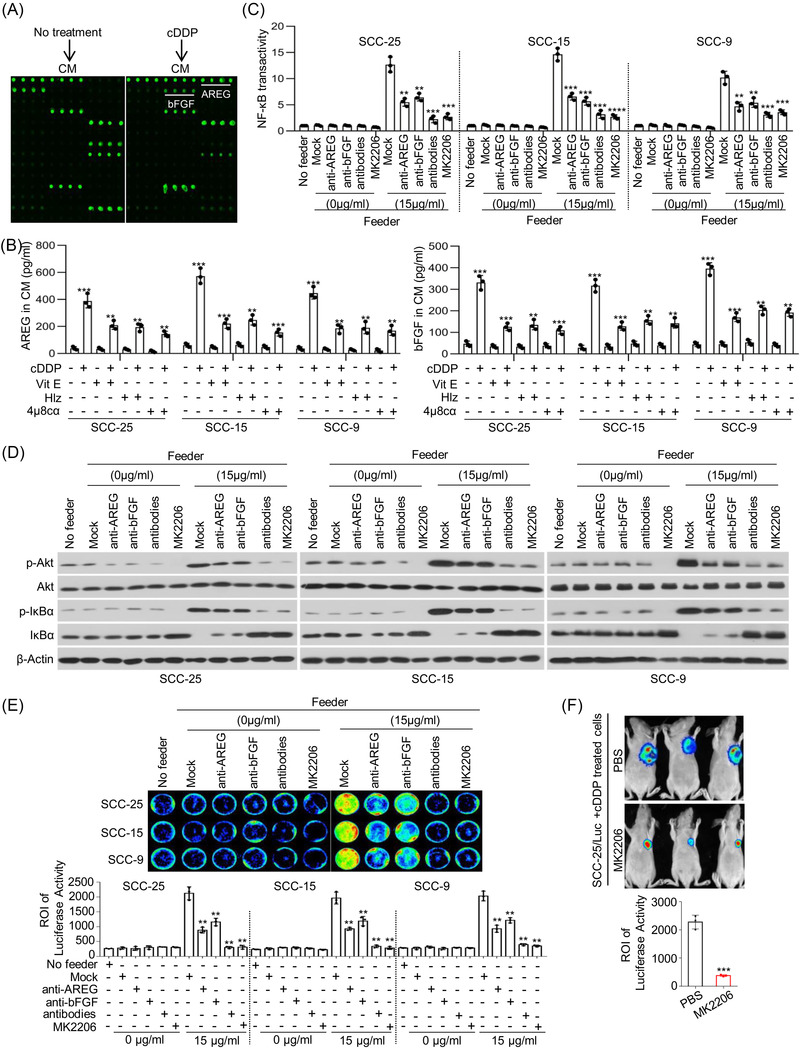
Cytotoxic treatment‐induced dying cells promoted tumour cell repopulation via secretion of amphiregulin (AREG) and basic fibroblast growth factor (bFGF). (A) SCC‐25 cells were treated with or without cisplatin (cDDP) (8 µg/ml) for 24 h, and the conditioned medium (CM) was collected. The differentially expressed cytokines in the CM were identified using a cytokine array. (B) SCC‐25, SCC‐15 and SCC‐9 cells were treated with cDDP in combination with vitamin E (VitE), hydralazine (Hlz) or 4µ8cα for 24 h. The CM from further incubation for 24 h was collected. The protein levels of AREG and bFGF in CM were determined by enzyme‐linked immunosorbent assay (ELISA) (*n* = 3), ^**^ *p* < .01, ^***^ *p* < .001. (C and D) SCC‐25, SCC‐15 and SCC‐9 cells were treated with cDDP for 24 h. Another new SCC‐25, SCC‐15 and SCC‐9 cells were cocultured with feeder cells combination with AREG‐neutralising antibody (1 µg/ml), bFGF‐neutralising antibody (1 µg/ml) and Akt inhibitor MK2206 (800 µM). Transactivation of NF‐κB signalling in mentioned cells was measured by Cignal Reporter Assays (*n* = 3), ^**^ *p* < .01, ^***^ *p* < .001, ^****^ *p* < .0001 (C). p‐Akt, Akt, p‐IκBα, IκBα and β‐Actin were detected by western blots (D). (E) SCC‐25, SCC‐15 and SCC‐9 feeder cells were treated with cDDP for 24 h. SCC‐25/Luc, SCC‐15/Luc and SCC‐9/Luc reporter cells were cocultured with feeder cells in combination with neutralising antibody against AREG or bFGF or Akt inhibitor MK2206 in 24‐well plates. Cancer cell repopulation in vitro was observed by luciferase activities, ^**^ *p* < .01. (F) SCC‐25 cells were treated with cDDP for 24 h. SCC‐25/Luc cells were injected subcutaneously together with cDDP‐treated SCC‐25 cells in nude mice to generate xenograft tumours. The mice were randomly grouped and received i.p. injection of MK2206 (300 mg/kg) or phosphate‐buffered saline (PBS) once every 3 days, and tumour growth was represented by luciferase levels, ^***^ *p* < .001. Data in (B), (C), (E) and (F) are represented as mean ± SEM. Statistical significance was determined by a two‐tailed Student's *t*‐test

To identify the effectors by which dying cells stimulate living tumour cell repopulation, we compared the secreted protein profiles in the CM of SCC‐25 cells with or without cDDP treatment using an antibody array (Figures 5A and S7A). A series of proteins with significant differential expression were identified (Figure S7B and Table S3). The Gene Ontology analysis showed that the differentially expressed proteins (DEPs) belonged to cytokines and growth factors (Figure S7C). Kyoto Encyclopedia of Genes and Genomes analysis of the DEPs was performed, and biological pathway analysis indicated that the DEPs were associated with PI3K‐Akt signalling and NF‐κB signalling, which is consistent with our abovementioned results (Figure S7D). Among the differentially expressed cytokines, we focused on AREG and bFGF to identify the cytokines responsible for stimulating tumour cell repopulation. The protein levels of AREG and bFGF in the supernatant of the SCC‐25 cells treated with cDDP were higher than those in the CM of the SCC‐25 cells without treatment, and this result was extended to the other cell lines (Figure 5B). As expected, VitE, Hlz or 4µ8cα treatment disrupted the increase in AREG and bFGF protein levels in the CM of the cell lines treated with cDDP (Figure 5B), suggesting that the increase in AREG and bFGF resulted from activation of the ROS‐lipid peroxidation–ER stress axis by cDDP treatment. Given that activation of NF‐κB signalling is responsible for tumour cell repopulation, to address whether AREG and bFGF are critical to activate NF‐κB signalling, we added neutralising Abs against AREG and bFGF alone or in combination to the coculture system containing reporter cells and feeder cells. The addition of these two neutralising Abs synergistically reversed the transactivation of NF‐κB signalling in reporter cells (Figure 5C). Additionally, our results indicated that AREG‐ and bFGF‐neutralising Abs synergistically abolished the increase in p‐Akt and p‐IκBα levels and the decrease in IκBα levels in reporter cell lines induced by feeder cells (Figure 5D). Biologically, AREG‐ and bFGF‐neutralising Abs synergistically inhibited reporter tumour cell repopulation induced by feeder cells in vitro (Figure 5E). Consistently, Akt activity restrained by MK2206 also reversed the transactivation of NF‐κB signalling (Figure 5C), increased the protein expression levels of p‐Akt and p‐IκBα, but decreased IκBα level (Figure 5D), and suppressed the tumour cell repopulation of reporter cells induced by feeder cells (Figure 5E). Moreover, results from an in vivo model indicated that Akt inhibitor significantly repressed the effect of feeder cells on reporter cell repopulation (Figure 5F). These data suggest the important role of Akt/NF‐κB signalling in tumour cell repopulation stimulated by AREG and bFGF from dying tumour cells.

### 3.6 Molecular events for tumour cell repopulation correlate with poor clinical outcome

**FIGURE 6:**
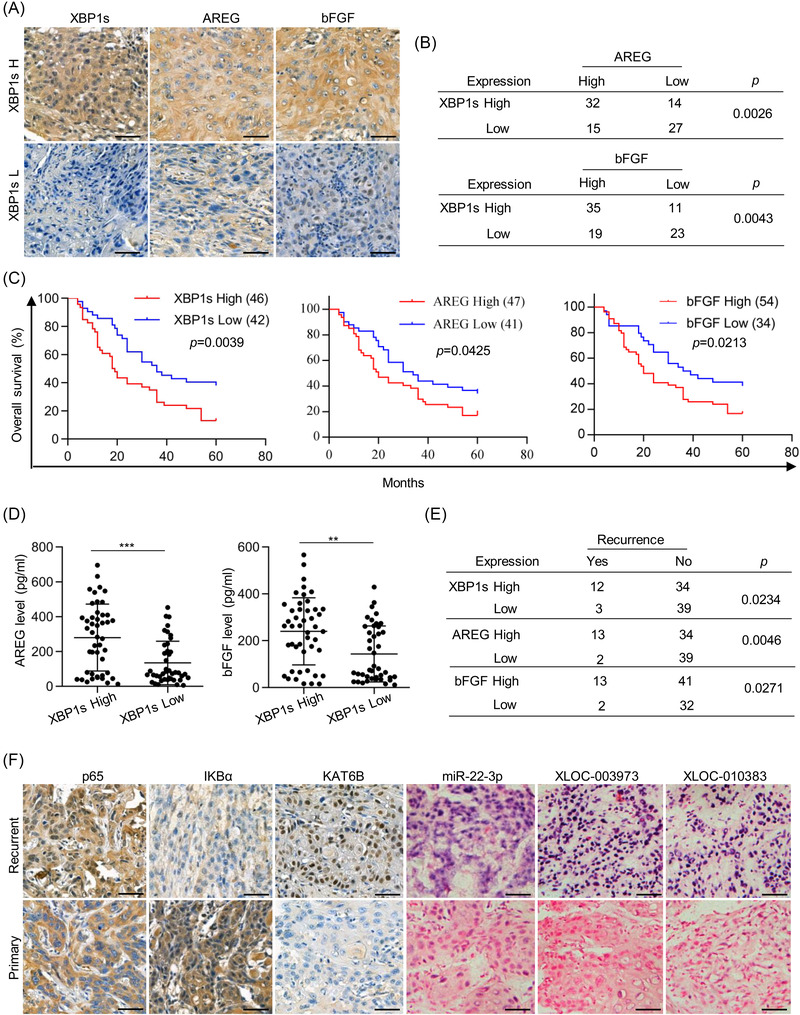
Molecular events for tumour cell repopulation correlate with poor clinical outcome. (A) The levels of X‐box binding protein‐1 (XBP1), amphiregulin (AREG) and basic fibroblast growth factor (bFGF) in representative cancer specimens were examined by immunohistochemistry (IHC) assays. Scale bar = 50 µm. (B) The correlation between the expression levels of XBP1s and AREG or bFGF was analysed. (C) The average IHC values of each protein (XBP1s, AREG, bFGF) in 88 tongue cancer tissues were obtained, and IHC scores above the average value indicated high expression, otherwise indicated low expression. Kaplan–Meier analysis indicated a correlation between XBP1s/AREG/bFGF levels and overall survival in patients with tongue cancer. Kaplan–Meier survival analysis is shown here with a two‐sided log‐rank *p*‐value. (D) The protein levels of AREG and bFGF in serum were examined by enzyme‐linked immunosorbent assay (ELISA) (*n* = 3), ^**^ *p* < .01, ^***^ *p* < .001. Data are presented as the mean value ± SEM. Statistical significance was determined by a two‐tailed Student's *t*‐test. (E) The correlation between the XBP1s/AREG/bFGF levels and tongue cancer recurrence was analysed. (F) Fifteen paired tissues of primary tongue cancer and recurrent tongue cancer were collected. The protein levels of p65, IκBα and lysine acetyltransferase 6B (KAT6B) were examined by IHC assays. The transcript levels of miR‐22‐3p, XLOC_003973 and XLOC_010383 were examined by in situ hybridisation (ISH) assays. Scale bars = 50 µm

Finally, we investigated the clinical role of molecular events induced by chemotherapy in human tongue cancer. The protein levels of XBP1s, AREG and bFGF were evaluated with immunohistochemical staining in the tissues from 88 tongue cancer patients who received cDDP‐based chemotherapy. A high XBP1s expression signal was detected in 46 tongue cancer tissues. Among the 46 tissues, 32 (69.57%) exhibited relatively high AREG expression and 35 (76.09%) exhibited relatively high bFGF expression (Figures 6A,B and S8A). However, AREG and bFGF protein levels were highly expressed in only 15 (35.71%) and 19 (45.24%) of 42 tongue cancer tissues with relatively low XBP1s expression, respectively (Figures 6B and S8A), suggesting a positive correlation between XBP1s and AREG and bFGF protein expression in tongue cancers. The tongue cancer patients with high XBP1s protein levels had a poorer overall survival than the patients with low XBP1s protein levels (Figure 6C). Similar results were obtained based on the levels of AREG and bFGF (Figure 6C). Moreover, high AREG and bFGF expression in tissues was well reflected in the serum (Figure 6D). Importantly, our investigation indicated that 12 out of the 46 (26.09%) tongue cancer patients with high XBP1s protein levels had recurrence, whereas only three out of the 42 (7.14%) patients with low XBP1s protein levels had recurrence (Figure 6E). We also found that tongue cancer patients with high AREG or bFGF were more likely to be recurrence (Figure 6E). Moreover, compared with primary cancer tissues, recurrent tissues showed higher p65 nuclear translocation and KAT6B protein levels but lower IκBα protein levels (Figures 6F and S8B), suggesting more activation of KAT6B/NF‐κB signalling in recurrent tissues. As expected, as an NF‐κB target, miR‐22 was highly expressed in recurrent tissues, accompanied by high expression of NF‐κB targets XLOC_003973 and XLOC_010383 to antagonise the effect of miR‐22 (Figures 6F and S8B). Furthermore, we also detected high expression of S100A8, PDGF and VEGF in tumor cells after eliminating AREG and bFGF once triggered (Figure S8C). This implied that once NF‐κB signal activation is triggered by feeder cells or NF‐κB activators, it can stimulate tumor cell repopulation. Collectively, these data indicate that ER stress triggered KAT6B‐dependent activation of NF‐κB signalling. This played a major role in cell death‐mediated cancer repopulation pathway.

## 4 DISCUSSION

**FIGURE 7:**
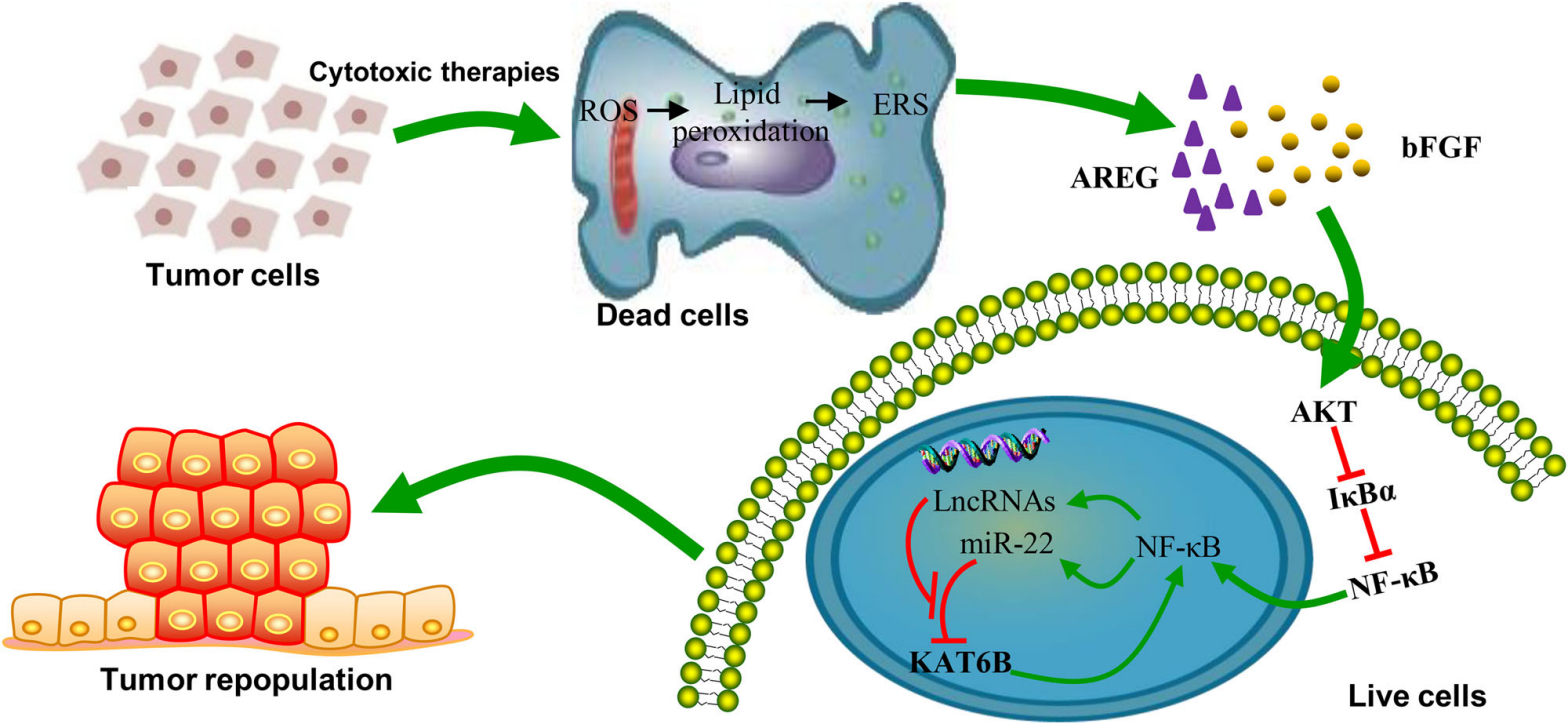
A graphical model of this study. A proposed model of the mechanisms by which cytotoxic therapies induce dead tumour cell‐mediated tumour cell repopulation

Tumour cell repopulation after anticancer therapies, which usually will inevitably lead to recurrence and metastasis, is the major cause of cancer‐related death. In response to cytotoxic therapies, there are both dead and live cells in the tumour. Elucidating the inextricable association between dead and live cells is critical to uncover the underlying mechanisms of tumour cell repopulation after cytotoxic therapies and to improve therapeutic efficacy. Here, our studies identified a novel mechanism responsible for dying tumour cell‐mediated tumour cell repopulation. We demonstrate that (1) in cytotoxic therapy‐induced dying tumour cells, ROS/lipid peroxidation–ER stress signalling triggers AREG and bFGF secretion to establish a population‐promoting extracellular environment. (2) Stimulated by dying cells, KAT6B‐dependent activation of NF‐κB signalling promotes reporter cell repopulation. (3) As NF‐κB targets, lncRNAs XLOC_003973 and XLOC_010383 disrupt miR‐22‐mediated downregulation of KAT6B expression, thereby enhancing KAT6B expression and finally leading to maintenance and amplification of KAT6B‐dependent NF‐κB signalling in reporter cells (Figure 7).

Our findings provide several insights into the molecular basis of tumour cell repopulation in both dying tumour cells and live tumour cells. First, we confirmed the direct association between cytotoxic treatment‐induced dying cells and tumour repopulation in tongue cancer. It seems paradoxical that cytotoxic treatment‐induced dying cells promote tumour repopulation, but these paradoxical observations have been well documented in experimental models and clinical investigations. Surgery remains the appropriate and potential cure for most cancer types, whereas surgery stress may enhance the risk of tumour recurrence and systemic metastasis.^22^, ^23^ Cytotoxic drugs provide long‐term clinical benefits to cancer patients but also potentially promote the disease progression of various types of malignancies by signals from apoptotic cells.^4^, ^14^, ^24^ These investigations and our findings strongly support the protective complex response to cytotoxic treatments exploited by tumours to preserve themselves from damage and to maintain tissue homeostasis. Second, we demonstrated that dying tumour cells promote the proliferation and growth of live tumour cells via ER stress induced by cytotoxic treatments. Although the association between dying and live tumour cells has been extensively observed, the molecular events in dying cells responsible for live tumour cell repopulation have rarely been investigated. The roles of caspase activation have been well explored. Caspase 3 activates the iPLA2‐arachidonic acid‐PGE2 axis and PKCδ/Akt/VEGF‐A axis and promotes apoptotic extracellular vesicle secretion enriched with various components of spliceosomes to enhance cell death‐induced tumour cell repopulation.^8^ However, our findings indicated that cell death‐mediated tumour cell repopulation was mainly dependent on ER stress triggered by cytotoxic treatment. These data are consistent with the literature showing that ER stress promoted intestinal Lgr5+ stem cell proliferation, crypt regeneration and survival in response to radiation‐induced intestinal injury.^25^ ER stress can be triggered by a variety of factors and regulated via diverse mechanisms. βarr1 overexpression decreased ER stress in radiation‐induced intestinal injury.^25^ TM9SF4 knockdown could enhance ER stress to sensitise chemoresistant breast cancer cells to drugs.^18^ Here, we found that cytotoxic treatment triggered ER stress via ROS‐mediated lipid oxidation, which is consistent with the literature indicating the link between ROS and ER stress.^26^ In particular, activation of the ER stress response factor XBP1 induced by lipid peroxidation in tumour‐associated DCs (tDCs) blunts antitumour immunity by inhibiting the capacity of tDCs to support antitumour T cells and then drives ovarian cancer progression.^27^ Third, concerning the direct mediators from dying cells that promote tumour cell repopulation, the cytokines AREG and bFGF were identified in this study, suggesting the critical roles of the paracrine niche created by dead cells. AREG, as an activating epidermal growth factor receptor ligand, is implicated in multiple cancer types and potently promotes malignant progression by promoting growth, invasion, metastasis, angiogenesis and therapeutic resistance.^28^ Several regulatory mechanisms for AREG expression by transcription factors and DNA methylation have been well described.^29^ Exposure to chemotherapy with cDDP could increase AREG expression and secretion in vivo and in vitro with unclear mechanisms.^30^ Consistently, we demonstrated that chemoradiotherapy could increase AREG protein levels in the conditioned culture medium of dying tumour cells. Mechanistically, increased AREG protein secretion resulted from ER stress triggered by chemoradiotherapy. Additionally, bFGF protein expression was upregulated in the conditioned culture medium of dying tumour cells via ER stress. The effects of bFGF on tumorigenesis and tumour growth by stimulating tumour cell proliferation and angiogenesis have been well documented.^31^, ^32^ Interestingly, bFGF secretion induced by UVB exposure from keratinocytes potentially promotes melanocyte proliferation, as we demonstrated that bFGF2 exerts an effect in a paracrine manner.^33^ Here, we found that AREG and bFGF2 from dying tumour cells synergistically promoted the proliferation of living tumour cells. Fourth, we revealed the molecular events responsible for tumour cell repopulation of living cells stimulated by dying tumour cells. The NF‐κB signal was markedly activated as a critical signalling pathway for tumour cell repopulation by conditioned culture medium or AREG and bFGF2 synergistically. The effect of NF‐κB signalling on tumour cell repopulation was dependent on the KAT6B protein. KAT6B (also known as MORF) is a member of the MYST family with histone acetyltransferase (HAT) activity. KAT6B acts as a transcriptional coactivator by acetylating histone H3 and H4 to achieve chromatin remodelling and thus is involved in stem cell self‐renewal^34^ and a variety of cancer types.^35^, ^36^ Inhibition of the HAT activity of KAT6B effectively induced tumour cell senescence and arrested tumour growth.^37^ In this study, ectopic overexpression of miR‐22 inhibited KAT6B expression in tongue cancer cells. As one of the most important tumour‐suppressive miRNAs depending on the molecular context, miR‐22 expression is downregulated in many cancer types. Downregulation of miR‐22 expression was correlated with tumour cell proliferation, invasion and metastasis.^38^, ^39^ Unexpectedly, rather than downregulation, miR‐22 expression was upregulated in living tumour cells incubated with CM of dying tumour cells. To address this issue, we sought to explore molecules that inhibit the effect of miR‐22 and focused on lncRNAs because of their roles as competing RNAs for miRNAs.^40^ Here, two lncRNAs (XLOC_003973 and XLOC_010383) were shown to potentially counter the effect of miR‐22 on KAT6B expression. Mutual inhibition between lncRNAs (XLOC_003973 and XLOC_010383) and miR‐22, as NF‐κB targets, led to upregulated KAT6B expression and subsequent feedback amplification of the NF‐κB signal, finally resulting in tumour cell repopulation.

Our findings have significant clinical implications. Currently, there is still no validated biomarker for predicting tongue cancer recurrence in the clinical setting. The data from the present study suggest that AREG and bFGF levels in patient tissue and serum might be explored as useful biomarkers predicting tumour cell repopulation and recurrence. Consistently, AREG levels in serum from hepatocellular carcinoma patients were higher than those in serum from a healthy population.^41^ bFGF expression is also significantly increased in the sera of multiple cancer types and is correlated with tumour recurrence.^42^ These data strongly suggest that AREG and bFGF can be valuable serological biomarkers. From a therapeutic point of view, our findings provide strong support for the rational design of novel treatment strategies against tumour cell repopulation by targeting several signalling pathways. Akt inhibition is effective in inhibiting cancer growth and progression. The selective Akt inhibitor MK2206 is currently under clinical trials in patients with several cancer types (clinical trial information: NCT01042379).^43^ Additionally, targeting AREG and bFGF will be effective in inhibiting tumour growth and preventing tumour recurrence. One strategy is to utilise neutralising Abs against AREG or bFGF to abrogate the triggering of related signalling. AREG‐neutralising antibody remarkably inhibited ovarian cancer growth and enhanced chemotherapy efficacy.^30^ Neutralising Abs against bFGF effectively impeded NSCLC development by suppressing tumour growth and angiogenesis.^42^

Recently, 3D bioprint tumor models or organoid models have become very suitable for exploring precision chemotherapy.^44^ In patient‐derived tumour organoids from human colorectal cancer, the researcher found that chemotherapy‐induced tumour cell death causes ATP release triggering P2X4 to promote live cancer cell survival in a mammalian target of rapamycin‐dependent manner.^45^ Maybe, we can use Abs against AREG or bFGF to abrogate the NF‐KB signalling triggered by chemotherapy or radiotherapy in tongue cancer organoid model in the future. Of course, the roles and mechanisms of AREG and bFGF in tumour cell repopulation and recurrence in cancer need further investigation. Approaches to disrupt signalling triggered by AREG and bFGF need further exploration.

## 5 CONCLUSIONS

In summary, we demonstrate that ROS‐lipid peroxidation–ER stress under cytotoxic therapy establishes an AREG‐ and bFGF‐based extracellular microenvironment of dying tumour cells, which activates KAT6B‐dependent NF‐κB signalling by disrupting the balance between miR‐22 and lncRNAs in live tumour cells to promote tumour cell repopulation. This study not only improved our understanding of the molecular mechanism responsible for tumour cell repopulation under cytotoxic therapy but also provided new avenues to identify valuable biomarkers predictive of therapeutic efficacy and facilitate a rational design of effective strategies to prevent tumour cell repopulation and recurrence.

## CONFLICT OF INTEREST

The authors declare they have no conflicts of interest.

## Supporting information

Supporting information
