# Supplementary material for: XBP1‐elicited environment by chemotherapy potentiates repopulation of tongue cancer cells by enhancing miR‐22/lncRNA/KAT6B‐dependent NF‐κB signalling
Source: Clin Transl Med. 2023 Jan 13;13(1):e1166. doi: 10.1002/ctm2.1166 (PMC9839876; doi:10.1002/ctm2.1166)
Supplement: Supplementary file 1 — Supporting information [file CTM2-13-e1166-s001.docx]

**XBP1-elicited environment by chemotherapy potentiates repopulation of tongue cancer cells via enhancing miR-22/lncRNAs/KAT6B dependent NF-kB signalling**

Xiaoting Jia^1,#^, Ge Wang^1,#^, Lihong Wu^2,#^, Hao Pan^3^, Li Ling^1^, Jianlei Zhang^1^, Qingquan Wen^1^, Jie Cui^1^, Zhimin He^1^, Bin Qi^1,^, Shuxu Zhang^1,*^, Liyun Luo^1,*^ and Guopei Zheng^1,*^

^1^Affiliated Cancer Hospital & Institute of Guangzhou Medical University; Guangzhou Municipal and Guangdong Provincial Key Laboratory of Protein Modification and Degradation; The State Key Laboratory of Respiratory, Guangzhou, Guangdong 510095 China.

^2^ Affiliated Stomatology Hospital of Guangzhou Medical University, Institute of Oral Disease, Guangzhou Medical University, Guangzhou, Guangdong, 510013 China.

^3^ Department of Periodontics & Oral Mucosal Section, Xiangya Stomatological Hospital & Xiangya School of Stomatology & Hunan Key Laboratory of Oral Health Research, Central South University, Changsha, 410013 China.

^#^ These authors contributed equally to the work.

***Corresponding Authors**:

Shuxu Zhang (gthzsx@163.com), Liyun Luo (1059619171@qq.com) and Guopei Zheng (zhengguopei@126.com), Affiliated Cancer Hospital & Institute of Guangzhou Medical University; Guangzhou Municipal and Guangdong Provincial Key Laboratory of Protein Modification and Degradation; The State Key Laboratory of Respiratory, Hengzhigang Road 78#, Guangzhou 510095, Guangdong, China. *Tel*: 86-020-83492353.


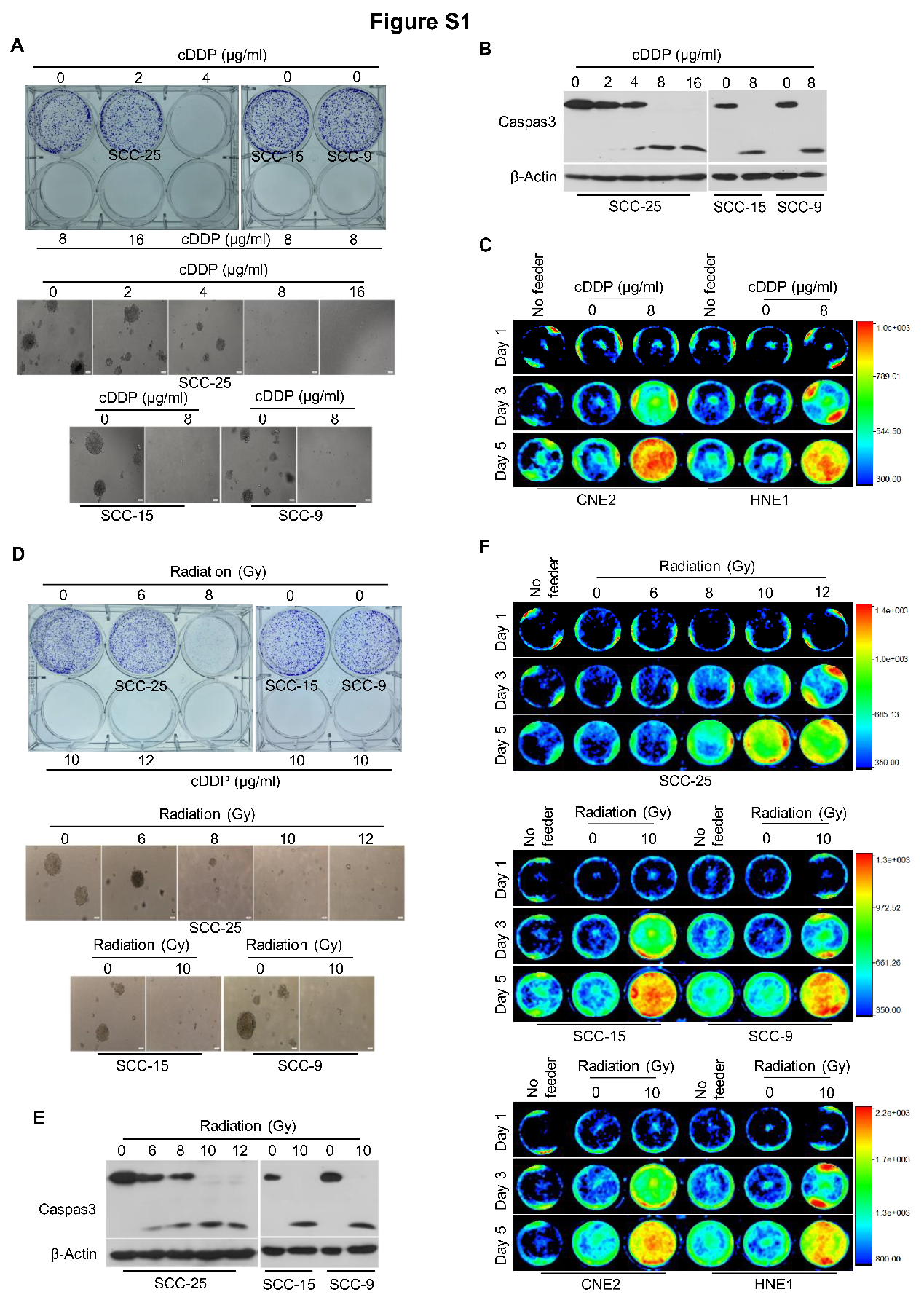


**Figure S1. Tongue cancer cell repopulation stimulated by cytotoxic treatment inducing dying cells.**

(A) Plate colony formation assays (upper) and soft agar colony formation assays (bottom) were performed to evaluate the colony growth of SCC-25, SCC-15 and SCC-9 cells under treatment with cDDP. (B) SCC-25, SCC-15 and SCC-9 cells were treated with cDDP at indicated concentrations for 24 hrs. The level of total Caspase3 and cleaved Caspase3 was examined by western blot assays. (C) CNE2/Luc and HNE1/Luc as reporter cells were seeded among respective feeder cells with or without cDDP treatment, or alone in 24-well plates. Cancer cell repopulation *in vitro* was observed by luciferase activities. (D) Plate colony formation assays (upper) and soft agar colony formation assays (bottom) were performed to evaluate the colony growth of SCC-25, SCC-15 and SCC-9 cells under treatment with radiation. (E) SCC-25, SCC-15 and SCC-9 cells were treated with radiation at indicated doses. The level of total Caspase3 and cleaved Caspase3 was examined by western blot assays. (F) SCC-25/Luc, SCC-15/Luc, SCC-9/Luc, CNE2/Luc and HNE1/Luc as reporter cells were seeded among respective feeder cells with or without radiation treatment, or alone in 24-well plates. Cancer cell repopulation *in vitro* was observed by luciferase activities. Data show a representative of three independent experiments.


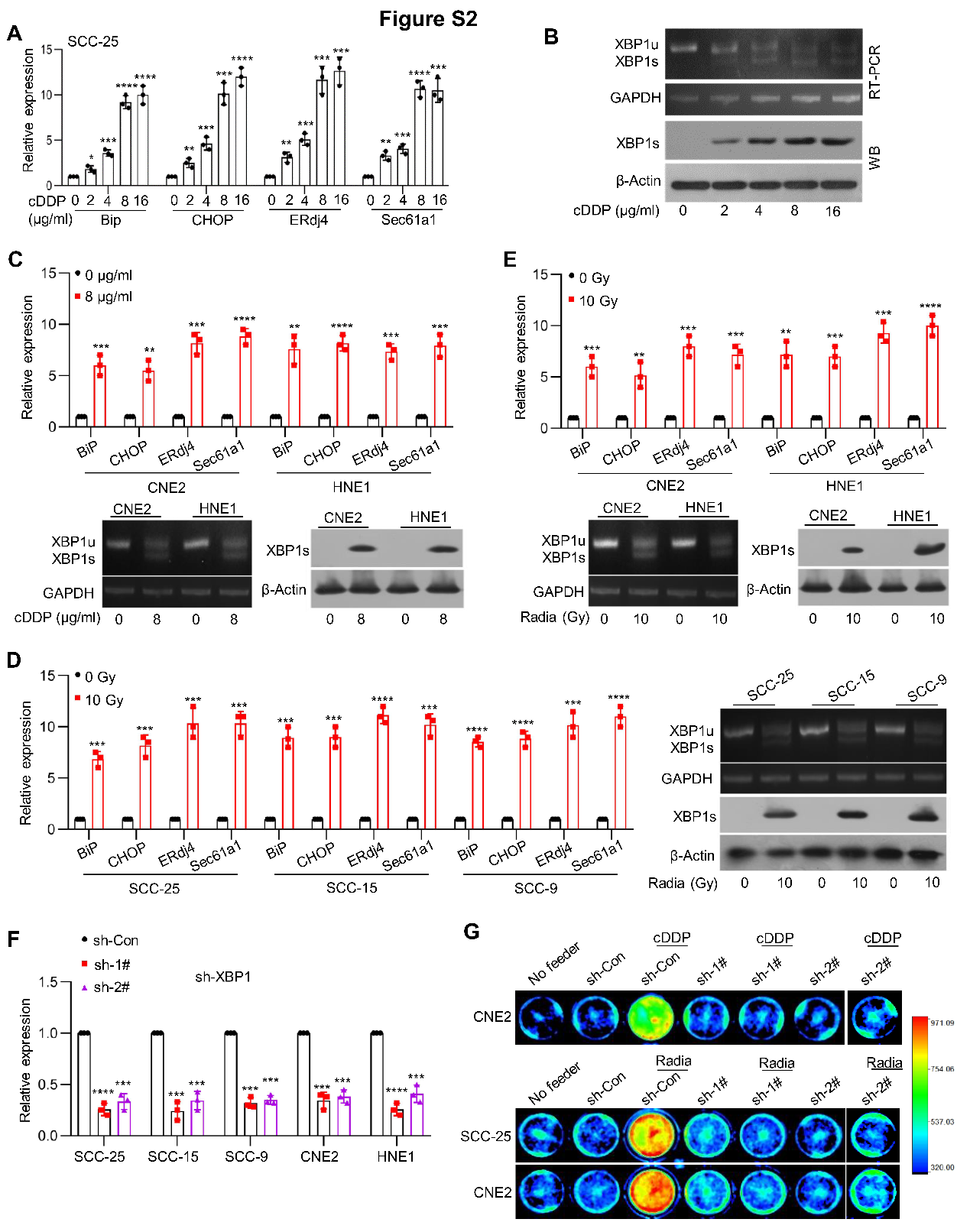


**Figure S2. Cytotoxic-treatment induced ER stress mediating tumour cell repopulation.**

(A and B) SCC-25 cells were treated with cDDP at indicated concentrations for 24 hrs. (A) The expression levels of Bip, CHOP, ERdj4 and Sec61a1 were measured by qRT-PCR (n=3), * *p*<0.05, ** *p*<0.01, *** *p*<0.001, **** *p*<0.0001. (B) XBP1 splicing was evaluated using RT-PCR (upper) and western blot (bottom). (C) CNE2 and HNE1 cells were treated with cDDP at indicated concentrations for 24 hrs. The expression levels of Bip, CHOP, ERdj4 and Sec61a1 were measured by qRT-PCR (n=3) (upper). XBP1 splicing was evaluated using RT-PCR (n=3) (bottom, left) and western blot (bottom, right), ** *p*<0.01, *** *p*<0.001, **** *p*<0.0001. (D and E) SCC-25, SCC-15, SCC-9, CNE2 and HNE1 cells were irradiated at 10 Gy. The expression levels of Bip, CHOP, ERdj4 and Sec61a1 were measured by qRT-PCR (n=3). XBP1 splicing was evaluated using RT-PCR and western blot. ** *p*<0.01, *** *p*<0.001, **** *p*<0.0001. (F) The efficiency of XBP1 knockdown using specific shRNAs was confirmed by qRT-PCR (n=3), *** *p*<0.001, **** *p*<0.0001. (G) Indicated reporter cells were seeded among respective feeder cells with or without XBP1 knockdown and cDDP treatment or radiation, or alone in 24-well plates. Cancer cell repopulation *in vitro* was observed by luciferase activities.


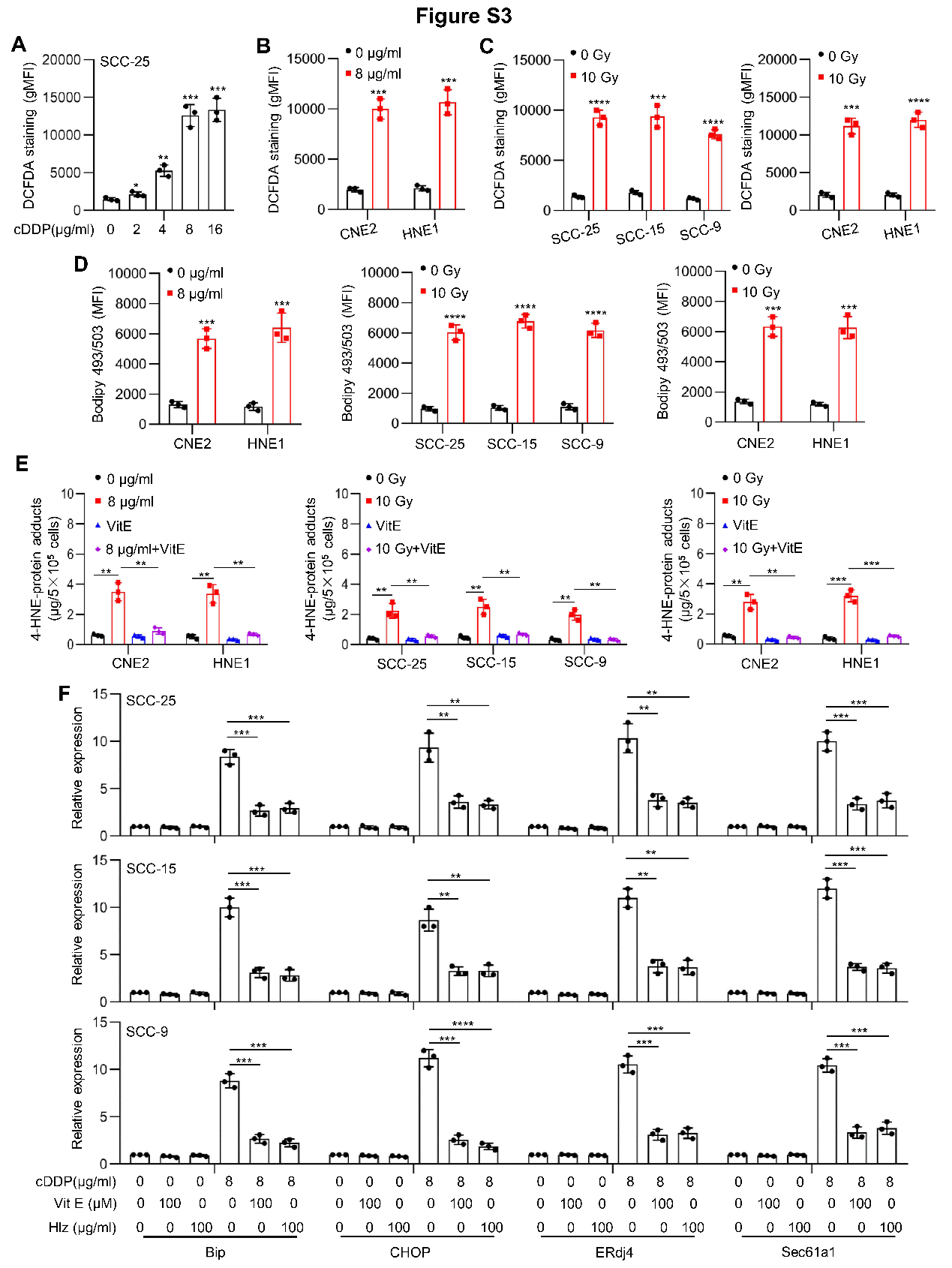


**Figure S3. Cytotoxic-treatment induced ER stress mediating tumour cell repopulation.**

(A-C) The intracellular ROS levels in indicated cells with cDDP treatment or radiation were evaluated using DCFDA staining (n=3). (D) The intracellular lipid content in indicated cells treated with cDDP or radiation were quantified using Bodipy 493/503 staining (n=3). (E) The intracellular levels of 4-HNE-protein adducts in indicated cells treated with cDDP or radiation in combination with ROS-scavenging agent vitamin E (VitE) were estimated via ELISA assay (n=3). (F) SCC-25, SCC-15 and SCC-9 cells were treated with cDDP in combination with VitE or hydralazine (Hlz). The expression levels of Bip, CHOP, ERdj4 and Sec61a1 were measured by qRT-PCR (n=3). * *p*<0.05, ** *p*<0.01, *** *p*<0.001, **** *p*<0.0001. Data are presented as mean values ± SEM. Statistical significance was determined by a two-tailed Student’s t test.

**
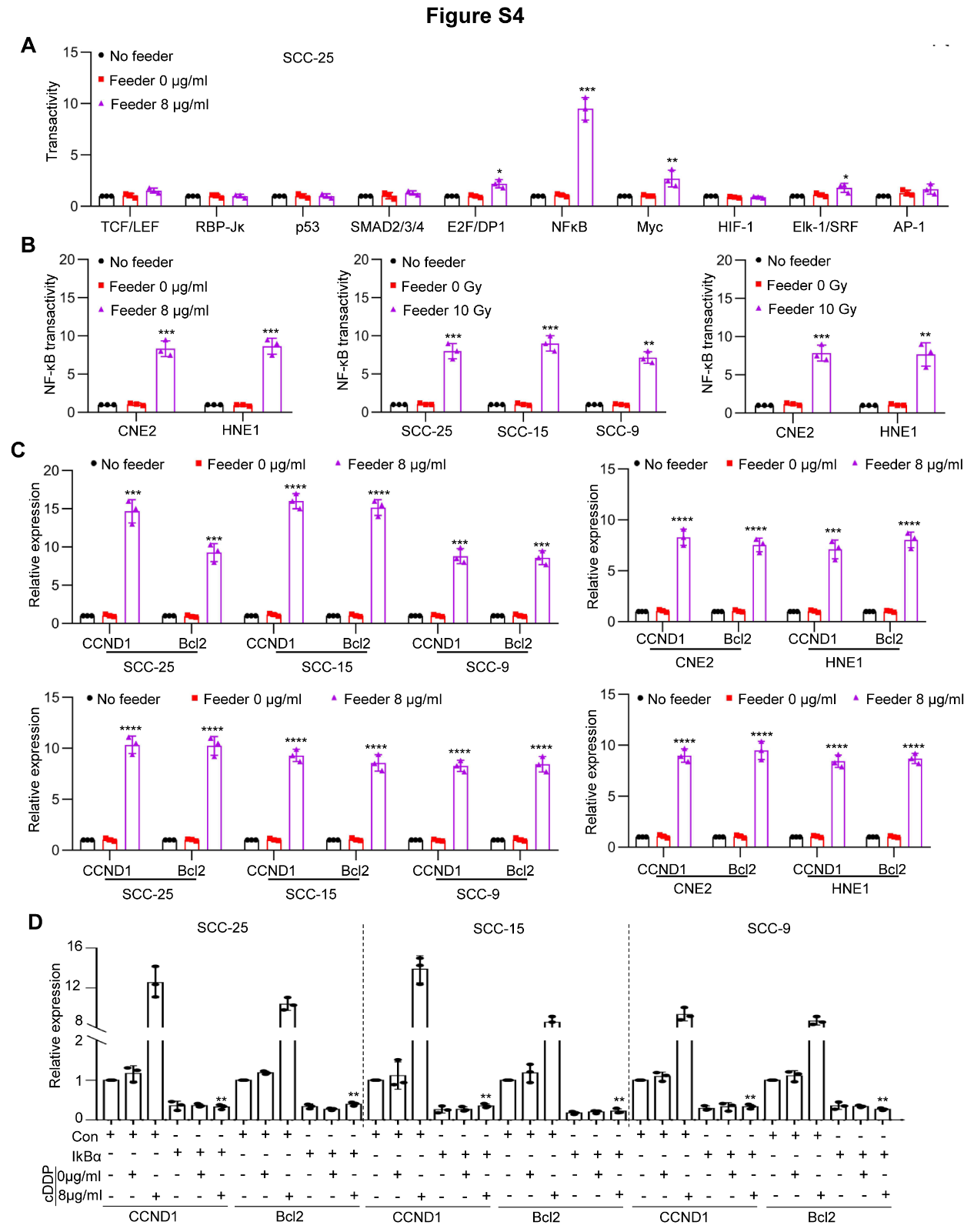
**

**Figure S4. KAT6B dependent NF-κB signalling responsible for living tumour cell repopulation stimulated by dying cells.**

1. The activities of transcription factors in SCC-25 cells after co-cultured with cDDP treated feeder cells were measured by Cignal Reporter Assays (n=3). (B) The transactivation of NF-κB signalling in indicated cells after co-cultured with cDDP or radiation treated feeder cells was measured by Cignal Reporter Assays (n=3). (C) The indicated cells were co-cultured with cDDP or radiation treated feeder cells. The mRNA levels of CCND1 and Bcl2 were detected by qRT-PCR (n=3). (D) SCC-25, SCC-15, and SCC-9 cells were treated with cDDP for 24h as feeder cells. Another new SCC-25, SCC-15, and SCC-9 cells were restored IκBα. Then, these tongue cancer cells with overexpressing IκBα cocultured with each feeder cells, respectively. The expression levels of CCND1 and Bcl2 in mentioned cells were evaluated by qRT-PCR assays (n=3). * *p*<0.05, ** *p*<0.01, *** *p*<0.001, **** *p*<0.0001. Data are presented as mean values ± SEM. Statistical significance was determined by a two-tailed Student’s t test.


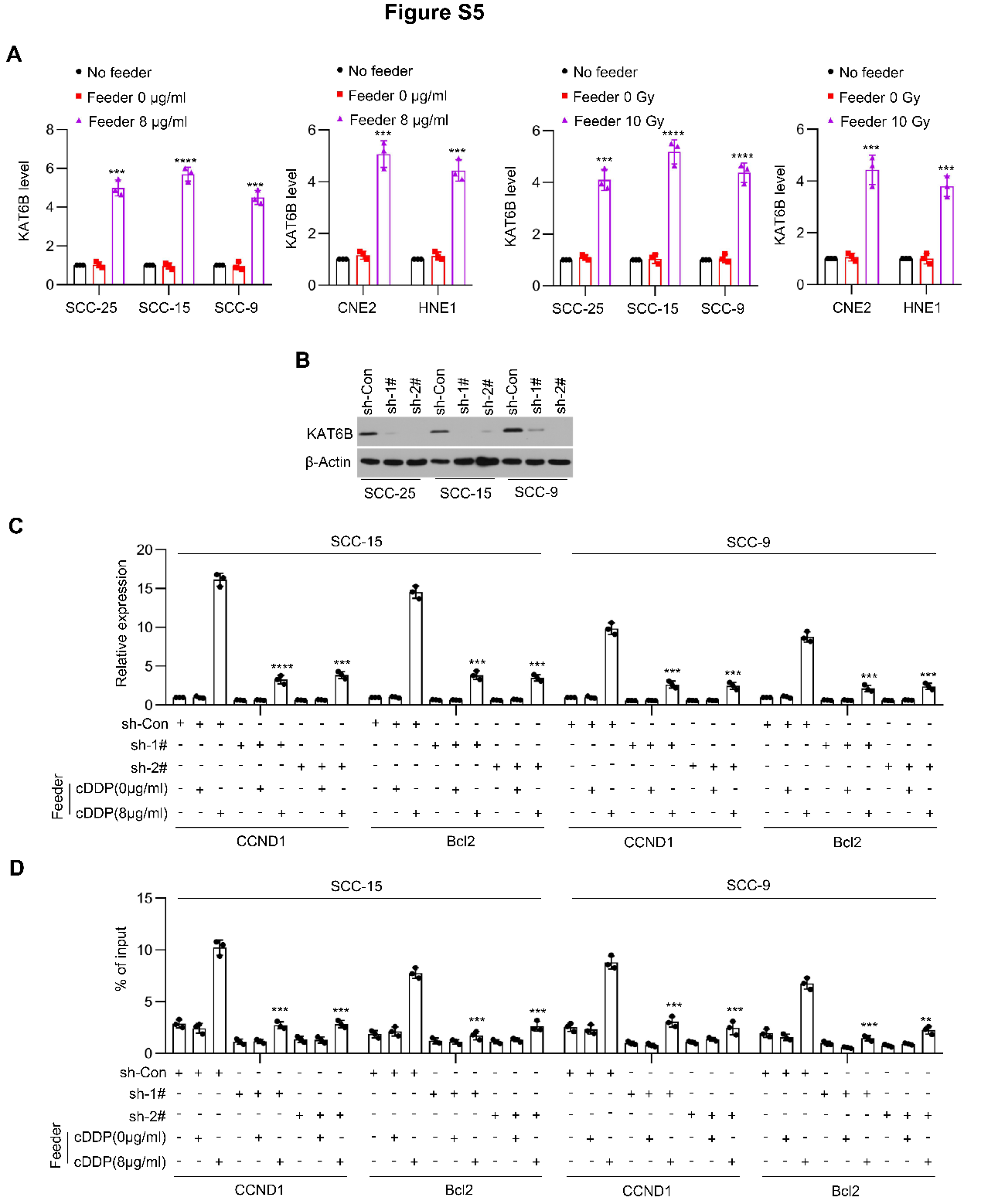


**Figure S5. KAT6B dependent NF-κB signalling responsible for living tumour cell repopulation stimulated by dying cells.**

1. The indicated cells were co-cultured with cDDP or radiation treated feeder cells. The mRNA level of KAT6B was detected by qRT-PCR (n=3). (B) SCC-25, SCC-15 and SCC-9 cells were transfected with specific shRNAs targeting KAT6B. The protein levels of KAT6B and β-Actin were detected by western blot. (C) SCC-15 and SCC-9 cells were transfected with shRNAs specifically targeting KAT6B and then co-cultured with cDDP treated feeder cells. The expression levels of CCND1 and Bcl2 were detected by qRT-PCR (n=3). (D) SCC-15 and SCC-9 cells were transfected with shRNAs specifically targeting KAT6B and then co-cultured with cDDP treated feeder cells. The H3K9Ac level at the promoter region of CCND1 and Bcl2 were detected by ChIP-qPCR (n=3). ** *p*<0.01, *** *p*<0.001, **** *p*<0.0001. Data are presented as mean values ± SEM. Statistical significance was determined by a two-tailed Student’s t test.


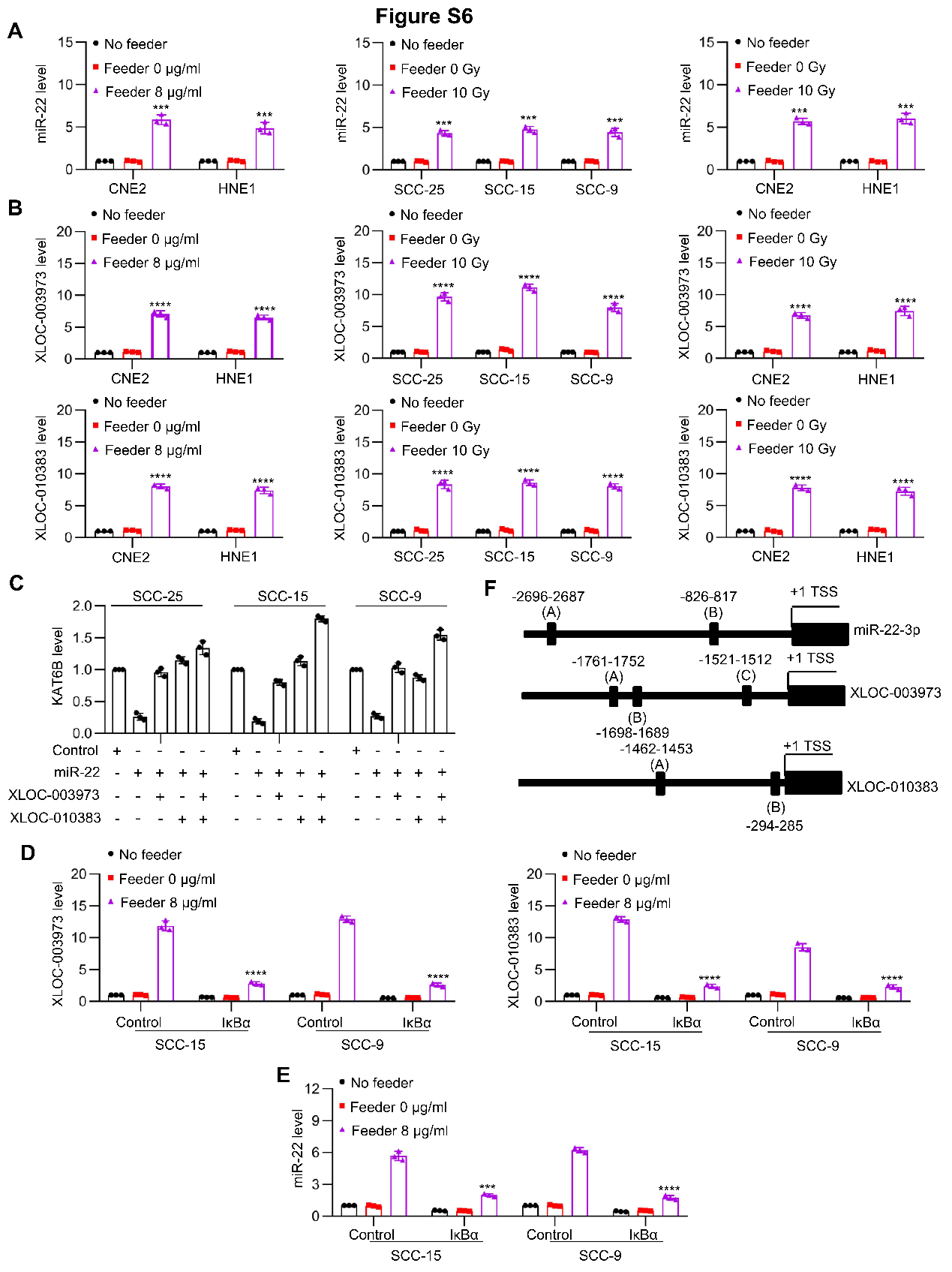


**Figure S6. Mutual confrontation between both NF-κB targets lncRNAs and miR-22 maintains KAT6B expression.**

(A) The indicated cells were co-cultured with cDDP or radiation treated feeder cells. The expression of miR-22 was measured by qRT-PCR (n=3). (B) The indicated cells were co-cultured with cDDP or radiation treated feeder cells. The expression of XLOC_003973 and XLOC_010383 was measured by qRT-PCR (n=3). (C) SCC-25, SCC-15 and SCC-9 cells were overexpressed by miR-22 in combination with XLOC_003973 or XLOC_010383. The mRNA level of KAT6B was measured by qRT-PCR (n=3). (D and E) SCC-15 and SCC-9 cells were transfected with pBabe-Con or pBabe-IκBα, and then co-cultured with cDDP treated feeder cells. The expression levels of XLOC_003973 and XLOC_010383 (D) and miR-22 (E) were measured by qRT-PCR (n=3). (F) A schematic representation of NF-κB binding sites within the 3kb putative promoter region of miR-22, XLOC_003973 or XLOC_010383. The first base of the precursor of miR-22 is defined as ‘+1’. The first base of the transcription start site of XLOC_003973 or XLOC_010383 is defined as ‘+1’. *** *p*<0.001, **** *p*<0.0001. Data are presented as mean values ± SEM. Statistical significance was determined by a two-tailed Student’s t test.


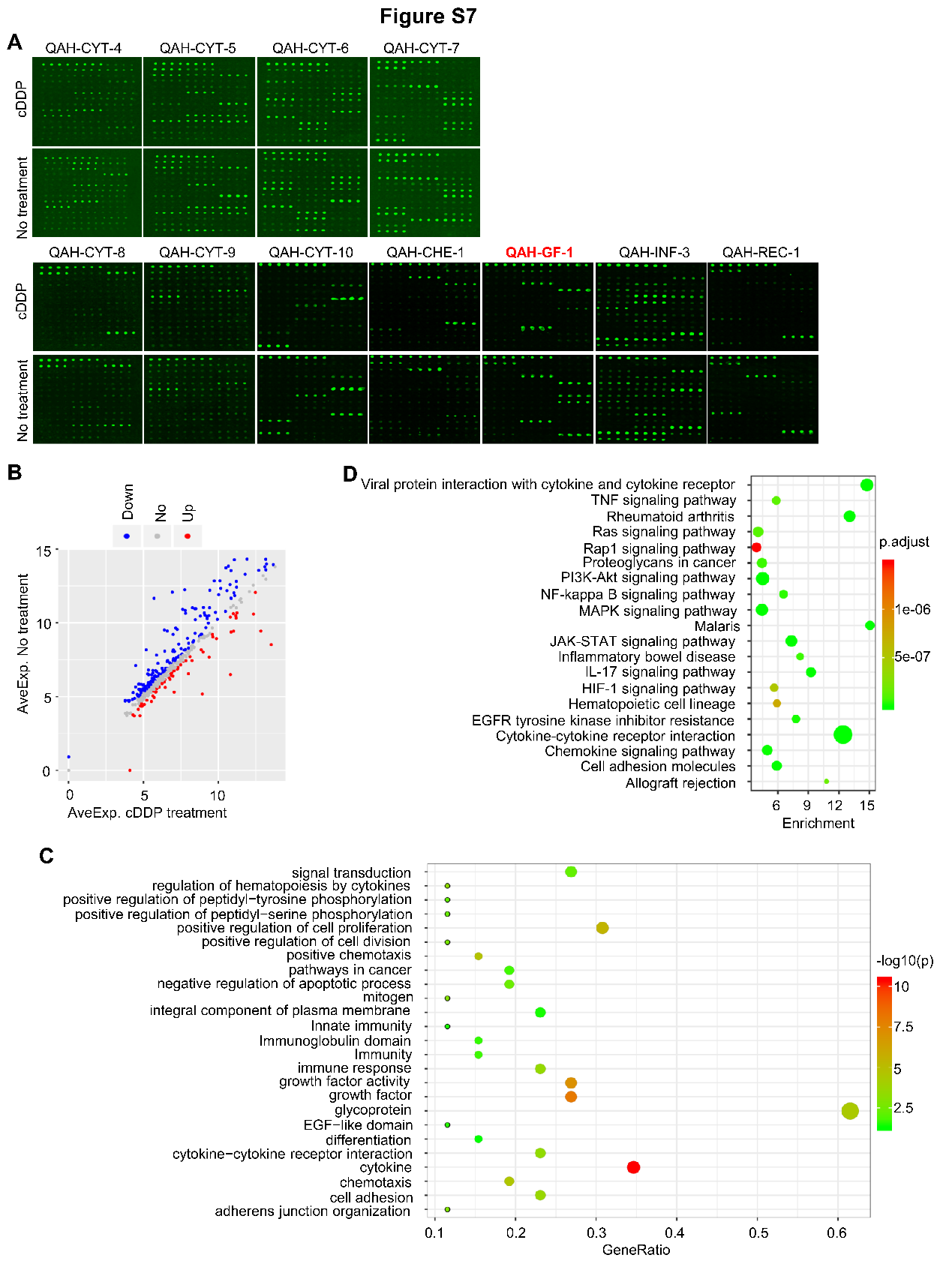


**Figure S7. Cytotoxic treatment induced dying cells promoted tumor cell repopulation via secretion of AREG and bFGF.**

(A) SCC-25 cells were treated by cDDP (8 μg/ml) for 24 hrs. The CM from further incubation for 24 hrs was collected. The differentially expressed cytokines in the CM were identified using cytokine array. (B) Scatter plot represents the differentially expressed proteins between CM from SCC-25 with and without cDDP treatment. (C) The GO analysis with the online tool DAVID was used to analyze the biological characteristics and function annotation of differentially expressed proteins in CM. (D) KEGG pathway analysis of differentially expressed proteins was performed using DAVID database.


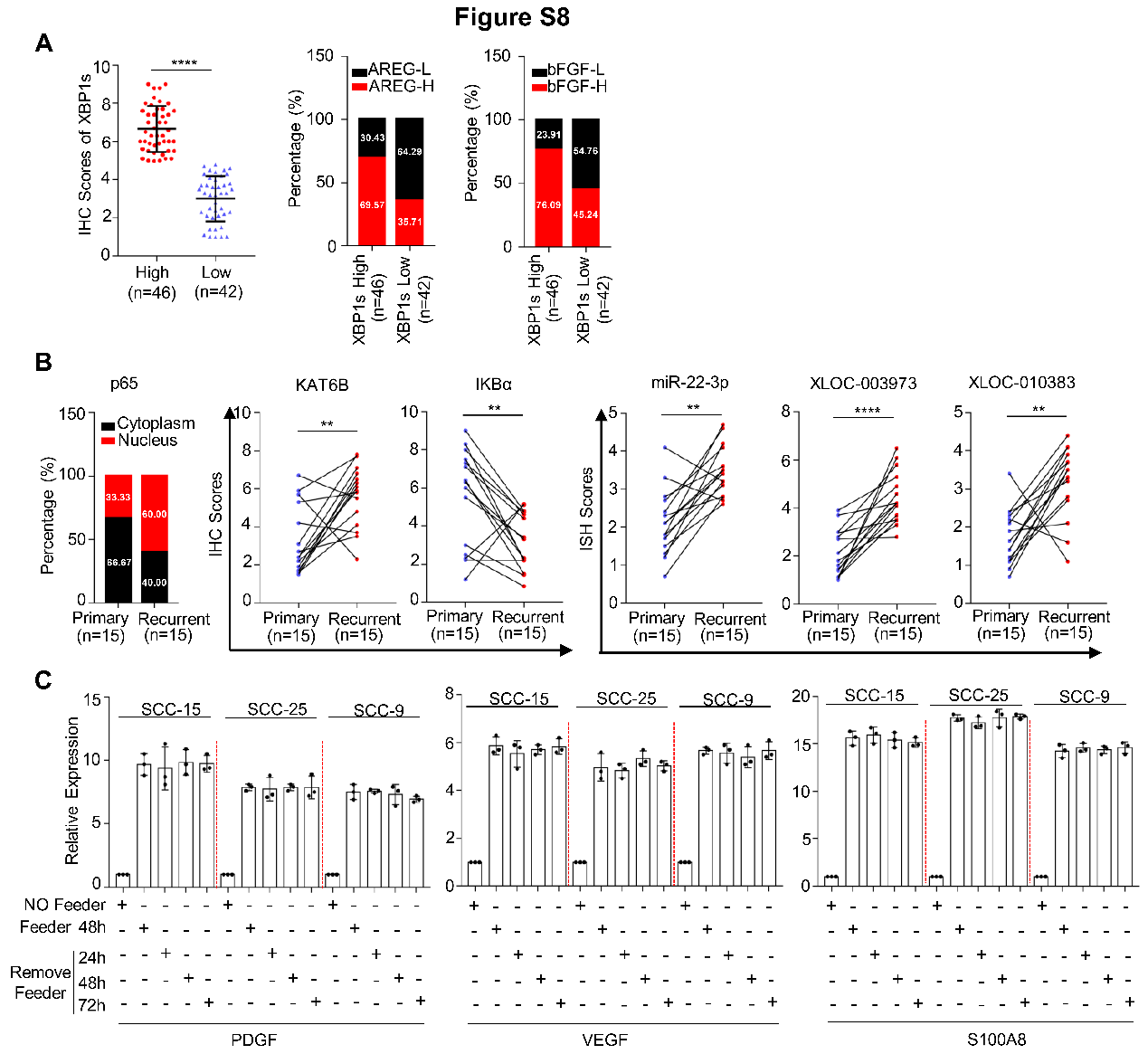


**Figure S8. Molecular events of tumour cell repopulation were analyzed in clinical tissues.**

(A) The levels of XBP1s, AREG and bFGF in representative cancer specimens were examined by IHC assays. (B) 15 paired tissues of primary and recurrent tongue cancer were collected. The protein levels of p65, IκBα and KAT6B were examined by IHC assays. The transcript levels of miR-22-3p, XLOC_003973 and XLOC_010383 were examined by ISH assays. (C) SCC-15, SCC-25 and SCC-9 cells were coculture with each cDDP-treated feeder cells for 48h, and then the feeder cells were removed, the living tumor cells were cultured for another 24h, 48h and 72h. qRT-PCR assays were performed to measure the expression levels of S100A8, PDGF and VEGF in mentioned cells (n=3). ** p<0.01, **** p<0.0001. Data are presented as mean values ± SEM. Statistical significance was determined by a two-tailed Student’s t test.

**Supplementary Table 1. Primers for RT-qPCR analyses of gene expression**

| **Gene** | **Forward (5' → 3')** | **Reverse (5' → 3')** |
| --- | --- | --- |
| XBP1 | CCCTCCAGAACATCTCCCCAT | ACATGACTGGGTCCAAGTTGT |
| Bip | CATCACGCCGTCCTATGTCG | CGTCAAAGACCGTGTTCTCG |
| CHOP | GGAAACAGAGTGGTCATTCCC | CTGCTTGAGCCGTTCATTCTC |
| ERdj4 | TCTTAGGTGTGCCAAAATCGG | TGTCAGGGTGGTACTTCATGG |
| Sec61a1 | TCATCCTGCCGGAAATTCAGA | AGGGTGATAGCGGTCCACA |
| CCND1 | GCTGCGAAGTGGAAACCATC | CCTCCTTCTGCACACATTTGAA |
| Bcl2 | GGTGGGGTCATGTGTGTGG | CGGTTCAGGTACTCAGTCATCC |
| KAT6B | GCCTTGCCTCCTATAAGGACC | TCCACATTGCGGAGATCATTAC |
| XLOC_00397 | CAACTGGCAGTGACAAGTGGT | CAGCTCTCAATTGCCATTTGGT |
| XLOC_010383 | CATTTTGGGTTGGGTGACAGC | ATCGGCAGATTTGAGCTTTCTTC |
| GAPDH | GGAGCGAGATCCCTCCAAAAT | GGCTGTTGTCATACTTCTCATGG |

**Supplementary Table 2. Primers for ChIP-qPCR analyses**

| **Gene** | **Forward (5' → 3')** | **Reverse (5' → 3')** |
| --- | --- | --- |
| miR-22-Site A | GAGCTATAGCGTCCCGCAGTC | GCATCTGGTCCGTGTAGTTCTC |
| miR-22-Site B | GCCATGCCCTTGGACTGAGTGT | TATGAATTCCAGGCTGTCTGCC |
| XLOC_003973-Site A | GAGGCGTTAAGTCCATTTACATTTAT | CACCACCAAGCTTGCCTTTCAAG |
| XLOC_003973-Site B | GTTAGTAGTTCCCTCAGGAGTTCTT | CCACAAGATATTTGGGACCAATATTCA |
| XLOC_003973-Site C | GAATATTGGTCCCAAATATCTTGTGG | GCAGGATGCTCCATGAGAAAATCAA |
| XLOC_010383-Site A | TCGCGGATCCTAAGAACCTTAGGAC | CTGTTCCCGGGCTGCGTTTGGA |
| XLOC_010383-Site B | GGACTGTGGGTATTAGGTGTCCAAG | GAATTCCCTCACCTGCCTTCTGCC |

**Supplement Table 3. Proteins with the most significant difference in cytokine assay**

| Protein ID | EX-SCC15cDDP | EX-SCC15Con | logFC | foldchange | regulation |
| --- | --- | --- | --- | --- | --- |
| TFPI | 5.711131 | 11.66449 | -5.95336 | 0.016138 | down |
| RBP4 | 6.925433 | 12.07124 | -5.14581 | 0.028246 | down |
| IL-6 | 13.54845 | 8.519832 | 5.028623 | 32.64121 | up |
| DKK-1 | 7.755434 | 12.23832 | -4.48289 | 0.044721 | down |
| AREG | 10.80334 | 6.486788 | 4.316554 | 19.92564 | up |
| VE-Cadherin | 4.087664 | 0 | 4.087664 | 17.00237 | up |
| bFGF | 8.944966 | 5.181874 | 3.763092 | 13.57699 | up |
| IL-8 | 12.85144 | 9.454225 | 3.397214 | 10.5357 | up |
| TIMP-4 | 7.608966 | 10.92383 | -3.31486 | 0.100491 | down |
| Angiogenin | 10.52448 | 13.72584 | -3.20136 | 0.108716 | down |
| MMP-10 | 11.08318 | 14.27664 | -3.19347 | 0.109313 | down |
| uPA | 10.67969 | 13.84064 | -3.16095 | 0.111804 | down |
| MMP-7 | 6.386676 | 9.515496 | -3.12882 | 0.114322 | down |
| IL-6R | 9.726288 | 12.8501 | -3.12381 | 0.11472 | down |
| TNF RI | 7.783309 | 10.8345 | -3.05119 | 0.120642 | down |
| Dkk-3 | 5.188362 | 8.18021 | -2.99185 | 0.125708 | down |
| IGFBP-2 | 6.592558 | 9.459974 | -2.86742 | 0.137032 | down |
| NSE | 12.35564 | 10.5932 | 1.762446 | 3.39273 | up |
| TGFa | 7.049537 | 5.310374 | 1.739164 | 3.338416 | up |
| GRO | 9.382421 | 7.681963 | 1.700458 | 3.25004 | up |
